# Analysis of Changes in Weight, Waist Circumference, or Both, and All-Cause Mortality in Chinese Adults

**DOI:** 10.1001/jamanetworkopen.2022.25876

**Published:** 2022-08-08

**Authors:** Yu Yuan, Kang Liu, Mengyi Zheng, Shuohua Chen, Hao Wang, Qin Jiang, Yang Xiao, Lue Zhou, Xuezhen Liu, Yanqiu Yu, Jiachen Wu, Xiong Ding, Handong Yang, Xiulou Li, Xinwen Min, Ce Zhang, Xiaomin Zhang, Meian He, Yan Zheng, Dianjianyi Sun, Lu Qi, Elena C. Hemler, Shouling Wu, Tangchun Wu, An Pan

**Affiliations:** 1 Department of Occupational and Environmental Health, Key Laboratory of Environment and Health, Ministry of Education and State Key Laboratory of Environmental Health (Incubating), School of Public Health, Tongji Medical College, Huazhong University of Science and Technology, Wuhan, China; 2 School of Public Health, Guangzhou Medical University, Guangzhou, China; 3 Graduate School of North China University of Science and Technology, Tangshan, China; 4 Health Department of Kailuan Group, Tangshan, China; 5 Department of Cardiovascular Diseases, Sinopharm Dongfeng General Hospital, Hubei University of Medicine, Shiyan, China; 6 State Key Laboratory of Genetic Engineering, Human Phenome Institute and School of Life Sciences, Fudan University, Shanghai, China; 7 Department of Epidemiology and Biostatistics, School of Public Health, Peking University Health Science Center, Beijing, China; 8 Department of Epidemiology, School of Public Health and Tropical Medicine, Tulane University, New Orleans, Louisiana; 9 Department of Nutrition, Harvard T.H. Chan School of Public Health, Boston, Massachusetts; 10 Department of Cardiology, Kailuan General Hospital, North China University of Science and Technology, Tangshan, China; 11 Department of Epidemiology and Biostatistics, Key Laboratory of Environment and Health, Ministry of Education and State Key Laboratory of Environmental Health (Incubating), School of Public Health, Tongji Medical College, Huazhong University of Science and Technology, Wuhan, China

## Abstract

**Importance:**

Although numerous studies have separately investigated the associations of changes in weight or waist circumference with mortality risk, few studies have examined the associations of concurrent changes in these 2 anthropometric parameters with all-cause mortality.

**Objective:**

To assess the associations of changes in body weight, waist circumference, or both, combined with all-cause mortality.

**Design, Setting, and Participants:**

This cohort study used data from 2 longitudinal cohort studies in Dongfeng-Tongji and Kailuan, China. Participants included 58 132 adults (aged 40 years and older) with measures of weight and waist circumference at baseline and follow-up visit. Statistical analysis was performed from June 2020 to September 2021.

**Exposures:**

Changes in weight and waist circumference between 2 visits (2008-2010 to 2013 in the Dongfeng-Tongji cohort, and 2006-2007 to 2010-2011 in the Kailuan study). Stable weight was defined as change in weight within 2.5 kg between the 2 visits and stable waist circumference was defined as changes within 3.0 cm. Changes were categorized as loss, stable, or gain for weight and waist circumference separately, and created a 9-category variable to represent the joint changes.

**Main Outcomes and Measures:**

All-cause mortality from follow-up visit (2013 in Dongfeng-Tongji cohort and 2010-2011 in Kailuan study) until December 31, 2018. Cox proportional hazard regression models were used to estimate the associations with adjustment for potential confounders. Results were obtained in the 2 cohorts separately and pooled via fixed-effect meta-analysis.

**Results:**

A total of 10 951 participants in the Dongfeng-Tongji cohort (median [IQR] age, 62 [56-66] years; 4203 [38.4%] men) and 47 181 participants in the Kailuan study (median [IQR] age, 51 [46-58] years; 36 663 [77.7%] men) were included in the analysis. During 426 072 person-years of follow-up, 4028 deaths (523 in the Dongfeng-Tongji cohort and 3505 in the Kailuan study) were documented. When changes in weight and waist circumference were examined separately, U-shape associations were found: both gain and loss in weight (weight loss: pooled hazard ratio [HR], 1.33; 95% CI, 1.23-1.43; weight gain: HR, 1.10; 95% CI, 1.02-1.19) or waist circumference (waist circumference loss: HR, 1.14; 95% CI, 1.05-1.24; waist circumference gain: HR, 1.11; 95% CI, 1.03-1.21) were associated with higher mortality risk compared with stable weight or waist group. When changes in weight and waist circumference were jointly assessed, compared with participants with stable weight and waist circumference (16.9% of the total population [9828 of 58 132] with 508 deaths), participants with different combinations of weight and waist circumference change all had higher mortality risks except for those with stable weight but significant loss in waist. Notably, those who lost weight but gained waist circumference (6.4% of the total population [3698 of 58 132] with 308 deaths) had the highest risk of all-cause mortality (HR, 1.69; 95% CI, 1.46-1.96; absolute rate difference per 100 000 person-years in the Dongfeng-Tongji cohort: 414; 95% CI, 116-819; and in the Kailuan study: 333; 95% CI, 195-492) among the joint subgroups.

**Conclusions and Relevance:**

In this cohort study, weight loss with concurrent waist circumference gain was associated with a higher mortality risk in middle-aged and older Chinese adults. This study’s findings suggest the importance of evaluating the changes in both body weight and waist circumference when assessing their associations with mortality.

## Key Points

**Question:**

What are the associations of changes in body weight, waist circumference, or both, with all-cause mortality?

**Findings:**

Using data from 2 cohort studies of 58 132 Chinese adults, this cohort study found U-shape associations of changes in weight and waist circumference with mortality risk. In addition, when changes in weight and waist circumference were jointly assessed, compared with participants who had stable weight (change ≤2.5 kg) and waist circumference (change ≤3.0 cm), those who lost more than 2.5 kg of weight but gained more than 3.0 cm of waist circumference had the highest risk of all-cause mortality among the joint subgroups.

**Meaning:**

Among middle-aged and older Chinese adults, people who lost weight but gained waist circumference had a much higher risk of mortality.

## Teaser

This cohort study assesses the associations of changes in body weight, waist circumference, or both, and all-cause mortality among Chinese adults aged at least 40 years.

## Introduction

The prevalence of obesity and its associated disease burden has become a global public threat.^1^ There has been a rapid increase in general obesity (measured by body mass index [BMI], calculated as weight in kilograms divided by height in meters squared) and abdominal obesity (measured by waist circumference) worldwide.^2,3^ The increasing rate in China was even faster, with an estimated 85 million adults aged 18 to 69 years in China who were obese in 2018, which was 3 times as many as in 2004.^4,5^ Mounting evidence has demonstrated the associations of excess BMI and/or waist circumference with all-cause mortality.^6,7,8^ However, it is widely acknowledged that weight and waist circumference could change over time,^9,10^ thus the association of changes in weight or waist circumference with mortality has attracted a lot of attention recently. A meta-analysis of 26 prospective studies (mostly in Western populations) has indicated that both weight gain and loss in middle-aged and older populations were associated with higher mortality risks,^11^ and similar findings were also reported in some studies in Asian populations.^12,13^ On the other hand, cohort studies have shown inconsistent findings for the association between changes in waist circumferences with mortality^14,15,16,17,18,19,20,21,22,23^: U-shape trend was reported in some studies,^16,20^ while positive association was reported with waist circumference gain,^14,21,22^ or waist circumference loss^15,23^ in some studies, and null association was reported in other studies.^17,18,19^

Despite the separate reports of the associations of changes in weight and/or waist circumference with mortality,^11,12,13,14,15,16,17,18,19,20,21,22,23^ few studies have investigated the simultaneous changes in body weight and waist circumference with mortality in large cohort studies. Moreover, although body weight and waist circumference generally change in the same direction at the population level,^10,24^ it is quite common that waist circumference gain could occur in people with stable weight or even in people who lose weight, particularly when people get older.^9,24,25,26^ Therefore, investigation of the concurrent changes in weight and waist circumference may further clarify the role of body shape changes with human health, and provide insights and guidelines for public health and clinical practice. To fill the knowledge gap, we investigated the associations of joint changes in weight and waist circumference with all-cause mortality in 2 longitudinal cohorts in China.

## Methods

### Study Population

Our study population was derived from 2 prospective cohorts: the Dongfeng-Tongji (DFTJ) cohort and the Kailuan study. Both studies are open cohorts (details shown in eFigure 1 in the Supplement) and were conducted in 2 large state-owned corporations the Dongfeng Motor Corporation (DMC), one of the largest auto manufacturers in Shiyan City of central China; and the Kailuan Company, one of the largest colliery companies in Tangshan City of northern China. The DFTJ cohort only recruited retired employees since 2008,^27^ and the Kailuan study recruited both active and retired employees since 2006.^28^ Between September 2008 and June 2010 (baseline, visit 0), all retired employees (n = 31 000) of the Dongfeng Motor Corporation were invited to participate in the DFTJ cohort. A total of 27 009 were enrolled (87% response rate), and among them, 24 175 participants completed examination in 2013 (visit 1, with a mean interval of 4.6 years). From July 2006 to October 2007 (baseline, visit 0), the Kailuan study enrolled a total of 101 510 participants from the Kailuan Company, and all participants received biennial physical examinations. To ensure a similar time interval in the 2 cohorts, we used data from the 2010 to 2011 survey (visit 2) in the Kailuan study for calculating the changes in weight and waist circumferences. A total of 68 746 participants attended both the baseline (2006-2007) and visit 2 (2010-2011) in the Kailuan study. In both cohorts, participants underwent questionnaire assessments, clinical examinations, and laboratory tests in both the baseline survey and follow-up visits. Vital status was updated annually.

We excluded participants with baseline age less than 40 years, or those with missing date, weight or waist circumference at the 2 visits, or those who had a history of cardiovascular disease (CVD), cancer, emphysema, bronchitis, active tuberculosis, or pregnancy at and during the 2 visits. Participants with extreme values of BMI less than 15 or greater than 50 at either visit were also excluded to reduce the probability of measurement error and influence of extreme values (and mostly unreliable values). Finally, a total of 10 951 participants in the DFTJ cohort and 47 181 participants in the Kailuan study remained in the current analyses. Detailed information regarding participant selection is available in eFigure 1 in the Supplement.

The DFTJ cohort was approved by the Ethics and Human Subject Committee of Tongji Medical College, Wuhan, China. The Kailuan study was approved by the Ethics Committee of the Kailuan General Hospital, Tangshan, China. All participants provided informed written consent. Our study follows the Strengthening the Reporting of Observational Studies in Epidemiology (STROBE) reporting guideline.

### Assessment of Changes in Weight and Waist Circumference

In each cohort, standing height and body weight were measured while participants were barefoot and wearing light indoor clothing using standard instruments and protocols.^27,29^ Meanwhile, waist circumference was measured with a soft nonstretchable tape at the midpoint between the lowest rib margin and the iliac crest. The changes in weight and waist circumference were calculated by subtracting the values obtained in the baseline survey from the values obtained during the follow-up visit in the DFTJ cohort (ie, from visit 0 to visit 1) and Kailuan study (ie, from visit 0 to visit 2). For the weight change analysis, we classified participants into the following categories: weight loss (lost >2.5 kg of weight), stable weight (change ≤2.5 kg), and weight gain (gained >2.5 kg), comparable to previous studies.^12,30^ For the waist circumference change analysis, we classified participants into 3 groups based on the distribution in 2 cohorts: waist circumference loss (lost >3.0 cm of waist circumference), stable waist circumference (waist circumference change ≤3.0 cm), and waist circumference gain (gained >3.0 cm), similar to previous studies.^17,31^

### Ascertainment of Outcomes

Both cohorts were conducted in large state-owned corporations, which have organized health care systems (including affiliated hospitals) and provided full coverage of medical insurance. Therefore, all participants could be tracked for mortality through review of medical insurance documents, social insurance records, hospital records, and death certificates. Deaths were followed until December 31, 2018.

### Assessment of Covariates

At baseline and subsequent follow-up visits in both cohorts, trained health workers administered a standardized questionnaire to collect information on socio-demographic characteristics (age, sex, and educational attainment), lifestyle behaviors (alcohol consumption, tobacco smoking, physical activity, and dietary intake), personal health and medical history (eg, cardiovascular disease, cancer, hypertension, diabetes). Additionally, participants underwent physical examinations. Details of data collection and covariates’ definitions are shown in the eAppendix in the Supplement.

### Statistical Analysis

Basic characteristics of the study population were described for each cohort as means (SDs) and percentages by category of variables. Cox proportional hazards models were used to estimate the hazard ratios (HRs) and 95% CIs for the associations of changes in weight and waist circumference with all-cause mortality. Changes in weight or waist circumference (categorical variables) were included as independent variables, and participants with stable weight or waist circumference were used as reference group. As participants are followed up prospectively for the occurrence of death, the effect of age needs to be tightly controlled because the incidence of death is strongly determined by age. Therefore, we used the acquired age (age at risk)^32^ during the study risk rather than the age at baseline that determines current risk. Multivariate models were stratified by age at risk (5-year interval),^32^ sex, and adjusted for height, smoking status (never, previous, or current smoker), alcohol consumption (never, previous, or current drinker), dietary pattern (favorable, intermediate, and unfavorable patterns), educational attainment (primary school or below, middle school, high school or beyond), and physical activity (hours per week in DFTJ, and no or occasional or regular physical activity in Kailuan study), diabetes status (yes or no), and hypertension status (yes or no). For the analysis of weight change, we additionally adjusted for weight at cohort recruitment and waist circumference change. For the analysis of waist circumference change, we adjusted for waist circumference at cohort recruitment and weight change. We categorized the changes as loss, stable, or gain for weight and waist circumference separately, and created a 9-category variable to represent the joint changes. In the analysis of joint changes in weight and waist circumference with all-cause mortality, participants with stable weight and waist circumference were set as the reference group, and similar covariates were adjusted in the models. We adjusted for the covariates collected at visit 1 in DFTJ and visit 2 in Kailuan, except for age and anthropometric measures at cohort recruitment.

We did all analyses separately in each cohort and pooled the cohort-specific HRs by both fixed-effect (main analyses) and random-effects (sensitivity analyses) models to obtain a summarized risk estimate. We further conducted exploratory analyses to identify potential risk factors of the changes in weight and waist circumference. More details of statistical methods including several stratified analyses (across baseline age, sex, BMI, waist circumference, diabetes, and physical activity strata), sensitivity analyses (among never smokers, excluding deaths within 2 years, and among participants with BMI <28 and free of diabetes), and exploratory analyses are shown in the eAppendix in the Supplement. The analyses were performed using SAS version 9.4 (SAS Institute) and Stata 14 (StataCorp) from June 2020 to September 2021. All *P* values were 2-sided, and statistical significance was defined as *P* < .05.

## Results

### Study Participants

A total of 58 132 participants (10 951 in the DFTJ cohort and 47 181 in Kailuan study) were included in the analysis. In the DFTJ cohort, the median (IQR) age was 62 (56-66) years, and 4203 (38.4%) were men. In the Kailuan study, the median (IQR) age was 51 (46-58) years, and 36 663 (77.7%) were men. Within a median (IQR) duration of 4.60 (4.58-4.62) years for the DFTJ cohort (2008-2010 to 2013) and 4.01 (3.76-4.34) years for the Kailuan study (2006-2007 to 2010-2011) between baseline and follow-up visits, participants experienced a median (IQR) of 0 (−2.5 to 3.0) kg in weight change and a 2 (−4.0 to 7.0) cm increase in waist circumference. Table 1 presents the baseline characteristics of the participants according to the categories of changes in weight and waist circumference in the 2 cohorts separately. At recruitment in the DFTJ cohort, the mean (SD) baseline weight was 62.7 (10.0) kg and waist circumference was 82.2 (9.2) cm; and at recruitment in the Kailuan study, baseline weight was 70.3 (10.8) kg and waist circumference was 87.4 (9.4) cm.

**Table 1.** Basic Characteristics of the Participants by Weight and Waist Circumference Change

Characteristics	No. (%)					
	Weight change, kg			Waist circumference change, cm		
	Loss (>2.5)	Stable (≤2.5)	Gain (>2.5)	Loss (>3.0)	Stable (≤3.0)	Gain (>3.0)
DFTJ cohort						
No. (%) of participants	3647 (33.3)	5477 (50.0)	1827 (16.7)	2519 (23.0)	4294 (39.2)	4138 (37.8)
Change duration, mean (SD), y	4.6 (0.1)	4.6 (0.1)	4.6 (0.1)	4.6 (0.1)	4.6 (0.1)	4.6 (0.2)
Age at cohort recruitment, mean (SD), y	62.6 (7.4)	61.1 (7.0)	60.5 (7.3)	61.7 (7.1)	61.4 (7.1)	61.5 (7.3)
Men	1580 (43.3)	1966 (35.9)	657 (36.0)	932 (37.0)	1658 (38.6)	1613 (39.0)
Women	2067 (56.7)	3511 (64.1)	1170 (64.0)	1587 (63.0)	2636 (61.4)	2525 (61.0)
Weight at cohort recruitment, mean (SD), kg	65.3 (10.1)	61.8 (9.6)	60.2 (9.9)	63.1 (10.2)	62.8 (10.1)	62.4 (9.9)
BMI at cohort recruitment, mean (SD)^a^	25.1 (3.2)	24.1 (3.1)	23.5 (3.2)	24.5 (3.2)	24.3 (3.1)	24.2 (3.2)
Height at cohort recruitment, mean (SD), cm	161.3 (7.7)	160.0 (7.4)	159.9 (7.3)	160.5 (7.5)	160.5 (7.6)	160.3 (7.5)
Waist circumference at cohort recruitment, mean (SD), cm	83.9 (9.4)	81.6 (8.9)	80.2 (8.9)	87.2 (9.2)	82.3 (8.5)	78.9 (8.3)
Physical activity, mean (SD), h/wk^b^	9.6 (7.4)	9.6 (7.4)	9.3 (7.6)	9.9 (7.6)	9.5 (7.4)	9.4 (7.3)
Smoking status^b^						
Never	2728 (74.8)	4307 (78.6)	1410 (77.2)	1963 (77.9)	3321 (77.3)	3161 (76.4)
Previous	345 (9.5)	454 (8.3)	171 (9.4)	199 (7.9)	357 (8.3)	414 (10.0)
Current	559 (15.3)	696 (12.7)	240 (13.1)	348 (13.8)	601 (14.0)	546 (13.2)
Alcohol intake^b^						
Never drinker	2623 (71.9)	3956 (72.2)	1304 (71.4)	1837 (72.9)	3111 (72.5)	2935 (70.9)
Former drinker	174 (4.8)	202 (3.7)	73 (4.0)	99 (3.9)	180 (4.2)	170 (4.1)
Current drinker	836 (22.9)	1298 (23.7)	448 (24.5)	573 (22.8)	990 (23.1)	1019 (24.6)
Dietary pattern^b^						
Daily FV, weekly but not daily meat	947 (26.0)	1364 (24.9)	440 (24.1)	672 (26.7)	1063 (24.8)	1016 (24.6)
Intermediate pattern	2199 (60.3)	3380 (61.7)	1098 (60.1)	1495 (59.4)	2602 (60.6)	2580 (62.4)
Less than daily FV, daily meat	468 (12.8)	683 (12.5)	262 (14.3)	331 (13.1)	585 (13.6)	497 (12.0)
Educational attainment^b^						
Primary school or below	1030 (28.2)	1455 (26.6)	423 (23.2)	743 (29.5)	1076 (25.1)	1089 (26.3)
Middle school	1379 (37.8)	2096 (38.3)	735 (40.2)	956 (38.0)	1663 (38.7)	1591 (38.5)
High school or beyond	1210 (33.2)	1896 (34.6)	655 (35.9)	798 (31.7)	1531 (35.7)	1432 (34.6)
Hypertension	2309 (63.3)	3387 (61.8)	1159 (63.4)	1567 (62.2)	2633 (61.3)	2655 (64.2)
Diabetes	856 (23.5)	884 (16.1)	325 (17.8)	481 (19.1)	844 (19.7)	740 (17.9)
Kailuan study						
No. (%) of participants	10 818 (22.9)	21 135 (44.8)	15 228 (32.3)	12 788 (27.1)	15 120 (32.1)	19 273 (40.9)
Change duration, mean (SD), y	4.0 (0.5)	4.1 (0.5)	4.1 (0.5)	4.1 (0.5)	4.1 (0.5)	4.1 (0.5)
Age at cohort recruitment, mean (SD), y	52.9 (9.2)	526 (8.8)	53.2 (9.0)	53.7 (9.3)	52.4 (8.9)	52.7 (8.7)
Men	8680 (80.2)	16 360 (77.4)	11 623 (76.3)	9748 (76.2)	11 746 (77.7)	15 169 (78.7)
Women	2138 (19.8)	4775 (22.6)	3605 (23.7)	3040 (23.8)	3374 (22.3)	4104 (21.3)
Weight at cohort recruitment, mean (SD), kg	75.2 (10.6)	69.9 (10.3)	67.4 (10.5)	70.6 (11.1)	70.3 (10.6)	70.1 (10.9)
BMI at cohort recruitment, mean (SD)^a^	26.6 (3.4)	25.1 (3.1)	24.2 (3.2)	25.4 (3.5)	25.2 (3.2)	25.0 (3.3)
Height at cohort recruitment, mean (SD), cm	168 (6.8)	166.9 (7.0)	166.9 (7.0)	166.8 (7.1)	167.1 (7.0)	167.4 (6.9)
Waist circumference at cohort recruitment, mean (SD), cm	89.4 (9.1)	87.1 (9.2)	86.3 (9.6)	93.2 (9.0)	87.5 (8.3)	83.4 (8.3)
Physical activity^b^						
No physical activity	3182 (29.4)	6569 (31.1)	4455 (29.3)	3720 (29.1)	4936 (32.7)	5550 (28.8)
Occasional physical activity	6003 (55.5)	11 172 (52.9)	8356 (54.9)	7267 (56.8)	7833 (51.8)	10 431 (54.1)
Regular physical activity	1616 (14.9)	3367 (15.9)	2397 (15.7)	1784 (14.0)	2326 (15.4)	3270 (17.0)
Smoking status^b^						
Never	6891 (63.7)	13 184 (62.4)	9820 (64.5)	8481 (66.3)	9346 (61.8)	12 068 (62.6)
Former	460 (4.3)	921 (4.4)	735 (4.8)	507 (4.0)	682 (4.5)	927 (4.8)
Current	3450 (31.9)	7004 (33.1)	4653 (30.6)	3785 (29.6)	5068 (33.5)	6254 (32.5)
Alcohol intake^b^						
Never drinker	7483 (69.2)	14 002 (66.3)	10 392 (68.2)	9127 (71.4)	9840 (65.1)	12 910 (67.0)
Former drinker	64 (0.6)	125 (0.6)	85 (0.6)	70 (0.6)	91 (0.6)	113 (0.6)
Current drinker	3253 (30.1)	6978 (33.0)	4726 (31.0)	3573 (27.9)	5160 (34.1)	6224 (32.3)
Dietary pattern (based on salt intake, g/d)^b^						
Favorable pattern (<6)	1849 (17.1)	3661 (17.3)	2359 (15.5)	2030 (15.9)	2528 (16.7)	3311 (17.2)
Intermediate pattern (6-9)	7996 (73.9)	15 285 (72.3)	11 242 (73.8)	9741 (76.2)	11 031 (73.0)	13 751 (71.4)
Unfavorable pattern (≥10)	955 (8.8)	2158 (10.2)	1605 (10.5)	1001 (7.8)	1532 (10.1)	2185 (11.3)
Educational attainment^b^						
Primary school or below	923 (8.5)	1803 (8.5)	1475 (9.7)	1150 (9.0)	1220 (8.1)	1831 (9.5)
Middle school	7962 (73.6)	15 299 (72.4)	11 095 (72.9)	9445 (73.9)	10 812 (71.5)	14 099 (73.2)
High school or beyond	1903 (17.6)	3992 (18.9)	2625 (17.2)	2175 (17.0)	3054 (20.2)	3291 (17.1)
Hypertension	5414 (50.1)	10 528 (49.8)	8001 (52.5)	6417 (50.2)	7584 (50.2)	9942 (51.6)
Diabetes	1685 (15.6)	2478 (11.7)	1646 (10.8)	1742 (13.6)	1834 (12.1)	2233 (11.6)

Abbreviations: BMI, body mass index; DFTJ, Dongfeng-Tongji; FV, fruit and vegetables.

^a^ BMI is calculated as weight in kilograms divided by height in meters squared.

^b^ Data were incomplete for these variables. In the DFTJ cohort, 0.2% (n = 23), 0.4% (n = 41), 0.3% (n = 37), 1.0% (n = 110), and 0.7% (n = 72) of participants had missing data for physical activity, smoking status, alcohol intake, dietary pattern, and educational attainment, respectively. In the Kailuan study, 0.1% (n = 64), 0.1% (n = 63), 0.2% (n = 73), 0.2% (n = 71), and 0.2% (n = 104) of participants had missing data for physical activity, smoking status, alcohol intake, dietary pattern, and educational attainment, respectively. The other variables included in the analyses did not have missing data. We did not present menopausal status in women given that nearly all women (n = 6725 [99.7%]) were postmenopausal in the DFTJ cohort and only a small proportion of women (n = 1007 [9.5%]) provided information of menopausal status in Kailuan study.

### Changes in Weight and Waist Circumference With All-Cause Mortality

During 426 072 person-years of follow-up, we documented 4028 deaths (523 in the Dongfeng-Tongji cohort and 3505 in the Kailuan study). Compared with the weight-stable group (change less than 2.5 kg), those with weight loss (lost more than 2.5 kg) or weight gain (gained more than 2.5 kg) had higher risks of all-cause mortality (weight loss: HR, 1.33; 95% CI, 1.23-1.43; weight gain: 1.10; 95% CI, 1.02-1.19) (Table 2). Meanwhile, compared with the waist circumference–stable group (change less than 3.0 cm), those with waist circumference loss (lost more than 3.0 cm) or gain (gained more than 3.0 cm) had higher risks of all-cause mortality (waist circumference loss: HR, 1.14; 95% CI, 1.05-1.24; waist circumference gain: 1.11; 95% CI, 1.03-1.21) (Table 3). Other variables included in Table 2 and 3 were shown in eTable 1 and 2 in the Supplement. U-shape dose-response associations were observed in both cohorts using the restricted cubic spline analysis (eFigures 2 and 3 in the Supplement).

**Table 2.** Associations Between Weight Change Categories and All-Cause Mortality

Variable^a^	Weight change, kg		
	Loss (>2.5)	Stable (≤2.5)	Gain (>2.5)
DFTJ cohort			
No. of events/person years	238/20 234	199/30 695	86/10 218
HR (95% CI)	1.56 (1.28-1.91)	1 [Reference]	1.30 (1.00-1.69)
Kailuan study			
No. of events/person years	949/83 503	1394/164 441	1162/116 981
HR (95% CI)	1.29 (1.18-1.40)	1 [Reference]	1.09 (1.00-1.18)
Pooled results			
No. of events/person years	1187/103 737	1593/195 136	1248/127 199
HR (95% CI)	1.33 (1.23-1.43)	1 [Reference]	1.10 (1.02-1.19)

Abbreviations: DFTJ, Dongfeng-Tongji; HR, hazard ratio.

^a^ The multivariable models were adjusted for height and weight at cohort recruitment, waist circumference change (continuous variables), smoking status, alcohol intake status, dietary pattern, educational attainment, physical activity, hypertension, and diabetes, and stratified by age at risk (5-year interval) and sex. We conducted cohort-specific analyses, which were pooled together using fixed-effect meta-analyses. The *P* for heterogeneity is 0.087 for the weight loss group, and it is 0.21 for the weight gain group.

**Table 3.** Associations Between Waist Circumference Change Categories and All-Cause Mortality

Variable^a^	Waist circumference change, cm		
	Loss (>3.0)	Stable (≤3.0)	Gain (>3.0)
DFTJ cohort			
No. of events/person years	141/14 017	184/24 032	198/23 098
HR (95% CI)	1.22 (0.97-1.54)	1 [Reference]	1.14 (0.92-1.41)
Kailuan study			
No. of events/person years	1102/98 141	991/117 698	1412/149 086
HR (95% CI)	1.13 (1.03-1.23)	1 [Reference]	1.11 (1.02-1.21)
Pooled results			
No. of events/person years	1243/112 158	1175/141 730	1610/172 184
HR (95% CI)	1.14 (1.05-1.24)	1 [Reference]	1.11 (1.03-1.21)

Abbreviations: DFTJ, Dongfeng-Tongji; HR, hazard ratio.

^a^ The multivariable models were adjusted for height and waist circumference at cohort recruitment, weight change (continuous variables), smoking status, alcohol intake status, dietary pattern, educational attainment, physical activity, hypertension, and diabetes, and stratified by age at risk (5-year interval) and sex. We conducted cohort-specific analyses, which were pooled together using fixed effect meta-analyses. The *P* for heterogeneity is 0.54 for the waist circumference loss group, and it is 0.82 for the waist circumference gain group.

The basic characteristics of participants according to the joint categories of weight and waist changes are shown in eTable 3 in the Supplement. The percentage of participants in the 9 joint groups ranged from 5.2% to 17.7%, and their associations with mortality are shown in the Figure. Specifically, compared with groups with stable weight and waist circumference (16.9% of participants [9828 of 58 132] with 508 deaths), those who maintained stable weight but gained waist circumference (17.7% of participants) had higher mortality risk (HR, 1.22; 95% CI, 1.08-1.37). All participants who gained weight (29.3% of participants) or lost weight (24.9% of participants) had significantly higher mortality risks, regardless of different waist circumference change status. Among the participants with weight gain, the adjusted HRs were 1.22 (95% CI, 1.04-1.44) for those with concurrent waist circumference loss, 1.20 (95% CI, 1.04-1.39) for those with stable waist circumference, and 1.26 (1.12-1.41) for those with waist circumference gain. Meanwhile, those who lost more than 2.5 kg of weight but gained more than 3.0 cm of waist circumference (6.4% of participants [3698 of 58 132] with 308 deaths) had the highest mortality risk (HR, 1.69; 95% CI, 1.46-1.96), and the mortality risk of this group was significantly higher than other groups. This group tended to have the highest BMI at cohort recruitment in both cohorts (eTable 3 in the Supplement). Among individuals with stable weight but more than 3.0 cm loss in waist circumference (11.2% of participants [6487 of 58 132]), the HR was 1.10 (95% CI, 0.97-1.26). Cohort-specific analyses yielded consistent findings, and the highest risks of all-cause mortality were consistently observed among those who lost weight but gained waist circumference (eFigure 4 in the Supplement).

**Figure.**
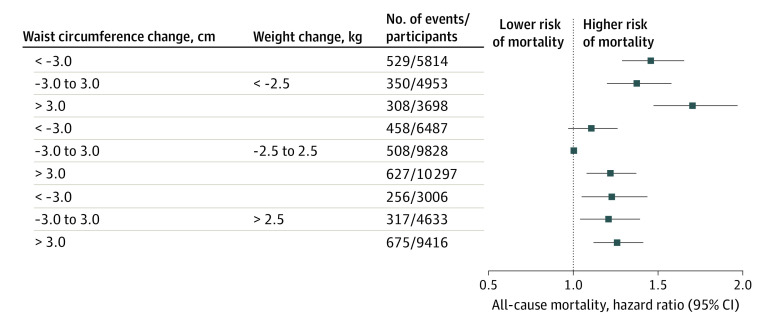
Adjusted Hazard Ratios for All-Cause Mortality Based on the Joint Changes in Weight and Waist Circumference The multivariable-adjusted model included the joint categories of weight and waist circumference changes, weight, height, and waist circumference at cohort recruitment, smoking status, alcohol intake status, dietary pattern, educational attainment, physical activity, hypertension, and diabetes; and stratified by age at risk (5-year interval) and sex. Cohort-specific results were pooled together using fixed-effect meta-analyses. Separate results for the DFTJ cohort and the Kailuan study are shown in eFigure 2 in the Supplement.

### Sensitivity Analyses

The association of weight change or waist circumference change with all-cause mortality were generally similar across age, sex, BMI, waist circumference, diabetes status, or physical activity level at cohort recruitment, and persisted among never smokers (eFigures 5 and 6 in the Supplement; eTables 4 and 5 in the Supplement). The heterogeneity statistics *I^2^* across cohorts were generally low (<50%), and results were not materially altered (eTables 6 and 7, eFigure 7 in the Supplement) when we used random-effects instead of fixed-effect models. The finding of highest risk among the weight loss and waist circumference gain group were observed in analyses excluding deaths occurred within 2 years after the follow-up visit (652 deaths were excluded; eFigure 8 in the Supplement), and in analyses across age, sex, baseline BMI, baseline waist circumference, and physical activity subgroups (eFigures 9-13 in the Supplement). We found significant interaction for baseline waist circumference (*P* for interaction = .01), whereas no significant interactions were found for other strata. Similar findings were observed in analyses among never smokers (eFigure 14 in the Supplement) and in analyses among participants with BMI less than 28 and without diabetes (eFigure 15 in the Supplement). This finding was evident among the 50 285 participants without diabetes, but not in the 7874 patients with diabetes (eFigure 16 in the Supplement), although the interaction test was insignificant.

### Exploratory Analyses

We further explored factors that were associated with changes in weight and waist circumference in both cohorts. Participants tended to lose weight and gain waist circumference with increasing age: participants lost a β (SE) of 0.31 (0.25-0.37) kg per 5 years in weight and gained a β (SE) of 0.48 (0.40-0.57) cm in waist circumference per 5 years in the DFTJ cohort; and lost 0.09 (0.06-0.12) kg per 5 years in weight and gained 0.61 (0.57-0.66) cm per 5 years in waist circumference in the Kailuan study (eTable 8 in the Supplement). Compared with men, women were more likely to lose weight (β [SE]: 0.47 kg [0.20-0.73 kg] in DFTJ; and β [SE]: 0.20 kg [0.04-0.36 kg] in Kailuan). Weight at baseline was positively associated with weight loss and waist circumference gain, whereas height and waist circumference at baseline were positively associated with weight gain and waist circumference loss. Diabetes history was positively associated with weight loss, whereas current smoking and low education attainment were positively associated with waist circumference gain. Because the group with weight loss and waist circumference gain had the highest mortality risk among the joint change groups, we further investigated factors that were related to this group (eTable 9 in the Supplement). Compared with the reference group of stable weight and waist circumference, people with older age, larger weight, shorter height, smaller waist circumference, and lower education attainment at baseline were more likely to be in this high-risk group in both cohorts.

## Discussion

In the 2 prospective cohorts of middle-aged and older Chinese adults, we found U-shape associations of changes in weight or waist circumference with risk of all-cause mortality. Compared with participants who had stable weight and waist circumference, those who maintained stable weight and gained waist circumference had significantly higher mortality risk among the joint subgroups. All participants who gained weight had higher mortality risks, regardless of the waist circumference change status. Notably, those who lost weight but gained waist circumference had the highest mortality risk. Participants with older age, larger weight, shorter height, smaller waist circumference, and lower education attainment at baseline were more likely to experience weight loss and waist circumference gain. These findings advance our understandings of the complex associations between changes in anthropometric measures and mortality, and they highlight the importance of maintaining body weight and waist circumference in middle-aged and older populations.

The association between changes in weight or BMI and all-cause mortality has been extensively explored in epidemiological studies.^11,12,13,23,30^ For example, a meta-analysis of 26 studies in middle-aged and older adults (mostly White individuals) reported that both weight loss (HR, 1.45; 95% CI, 1.34-1.58) and weight gain (HR, 1.07; 95% CI, 1.01-1.13) were associated with higher risks of all-cause mortality.^11^ Several studies in Asian populations, including Japanese^12^ and Singapore Chinese,^13^ also reported similar findings. Therefore, our finding of a U-shape association between weight changes and mortality is in agreement with those studies. Although studies have consistently demonstrated that central obesity, measured by evaluated waist circumference, is positively associated with mortality,^8^ the association between changes in waist circumference and mortality remains controversial.^14,15,16,17,18,19,20,21,22,23^ In our study, we found both waist circumference loss and waist circumference gain were associated with higher risks of all-cause mortality. In line with our findings, 2 other studies with small sample size (n = 627 in one study^16^ and n = 1061 in the other study^20^) also observed a U-shape trend of waist circumference change with mortality risk. However, due to the limited sample size, the results were not statistically significant for waist circumference gain groups in these studies.^16,20^ Meanwhile, 3 studies in European populations (Nordic women,^14^ Danish middle-aged participants,^21^ and UK adults^22^) suggested that substantial waist circumference gain (5 cm or more) was associated with higher mortality risk, but not waist circumference loss.^14,21,22^ By contrast, a cohort study in Australia reported that compared with participants with waist circumference gain of 1 to 5 cm, those with waist circumference change less than 1 cm (including waist circumference–loss and waist circumference–stable participants) had a higher risk of all-cause mortality, but not those with waist circumference increase of more than 5 cm. The Guangzhou Biobank Cohort with 17 773 Chinese adults reported that those with waist circumference loss (lost 5%), but not waist circumference gain (gained >5%), had a higher risk of all-cause mortality.^23^ Several studies examined the association of changes in waist circumference and mortality but the findings were not statistically significant.^17,18,19^ Nonetheless, the majority of these studies did not comprehensively exclude participants with cancer, cardiovascular disease or other chronic wasting diseases,^14,15,16,17,18,19,20^ which could cause potential reverse-causation bias.^33^ Participants with pre-existing diseases tend to lose waist circumference and have high case fatality rate,^33^ and the observed association between waist loss with mortality could be overestimated. Furthermore, the relatively small sample sizes (<3000),^14,16,17,18,20^ use of self-reported weight and waist circumference,^19,21^ long duration of changes (approximately 10 years),^15,19^ and lack of adjustment for potential confounding factors (ie, weight change)^14,15,16,17,18,19,23^ may lead to the inconsistent findings in previous studies. In our study, to reduce the reverse causation bias, in addition to excluded participants with serious illness, we also adjusted smoking to control the impact of confounding given that smokers tend to weigh less than nonsmokers while having much higher mortality rate.^34^ We further restricted the analyses in nonsmokers to estimate the mortality risk for weight change or waist circumference change, and the results did not change materially. Moreover, we excluded deaths in the first 2 years of follow-up to reduce bias due to reverse causation, and similar results were observed. However, it may not be possible to completely eliminate the reverse causation.

Changes in weight and waist circumference are most likely to occur in the same direction. However, this might not always happen, particularly in middle-aged and older individuals who experience body fat redistribution.^9^ Notably, we observed that increasing age was associated with weight loss and concurrent waist circumference gain in middle-aged and older Chinese adults. Weight loss in an aging population has been strongly linked to loss of lean mass^25^ while an elevation of waist circumference likely indicates either a gain in total body fat or abdominal body fat redistribution.^9^ However, previous studies^14,15,16,17,18,19,20,21,23^ have not explored the health consequences of joint changes in weight and waist circumference. The only study that simultaneously examined changes in the 2 measures was the EPIC-Norfolk study with participants aged 39 to 79 years in the UK, and Mulligan et al^22^ reported that weight loss with concurrent waist circumference gain was associated with highest mortality risk in men (n = 5469), whereas weight loss with stable waist circumference was associated with highest mortality risk in women (n = 6868). Nevertheless, the results were not available for the total population and never smokers, and there was no validation cohort.^22^ In addition, the number of participants who experienced weight loss with concurrent waist circumference gain was small in men (35 participants with 19 deaths, data not shown for women). With a much larger sample size in the current analysis, we observed consistent findings among men and women, as well as different age groups in 2 cohorts.

Although prior studies have separately explored the risk factors for changes in weight^35^ or waist circumference,^35,36^ to our knowledge, no study has specifically examined risk factors for the joint changes in weight and waist circumference. We addressed this knowledge gap and identified that age, education level, baseline weight, height, and waist circumference were important risk factors for the weight gain with simultaneous waist circumference loss phenotype. Therefore, special attention is needed for middle-aged and older adults with these characteristics, given that this body size change group had the highest mortality risk during the follow-up. Our findings may have important clinical implications because the potential health risks of this unique phenotype (weight loss with concurrent waist circumference gain) are not well studied and appreciated. The exact mechanisms underlying the unique phenotype remain to be further elucidated. The reduction of skeletal muscle mass (major constituent of lean mass) as well as muscle function, may contribute to reduced physical capability, impaired cardiopulmonary performance, unfavorable metabolic effects, falls, and disability.^26^ In addition, central obesity is associated with visceral fat accumulation and adverse metabolic profiles (hypertriglyceridemia, inflammation, insulin resistance, glucose intolerance, endothelial dysfunction), which result in higher cardiometabolic risk.^37^ Therefore, it is plausible that simultaneous loss of body weight and increase in central adiposity would lead to a much higher risk of mortality. Given that the process of losing lean mass and gaining adiposity with age is often ignored by individuals and the public, our study provides novel evidence to inform clinical guidelines and targeted prevention strategies for this high-risk group of individuals. Taken together, our findings underscore the importance of incorporating measures of changes in central adiposity in addition to changes in body weight.

Our study has various strengths. To enhance the external validity of the results, we used data from 2 well-established prospective cohorts in China. Despite the difference in some characteristics, these 2 cohorts generated consistent findings, indicating the robustness of our findings. Furthermore, our weight and waist circumference measurements were assessed by trained researchers, instead of self-reports, which reduced the misclassification bias from self-reported measures. Our repeated measurements of weight and waist circumference reflected the longitudinal changes over time. Additionally, to limit potential confounding by pre-existing chronic conditions and smoking,^6^ we excluded participants with cardiovascular disease, cancer, and chronic wasting diseases, and further conducted sensitivity analyses among never smokers. Finally, we are among the first studies to explore risk factors associated with concurrent changes in weight and waist circumference.

### Limitations

This study has some limitations. First, we did not directly measure lean muscle mass and fat mass, which is necessary in future studies. Second, we were not able to distinguish between intentional and unintentional changes in weight and waist circumference, although intentional weight loss has been associated with lower risk of mortality.^33^ Furthermore, there are differences in population characteristics between the 2 cohorts, but the consistent findings in the 2 cohorts suggest the generalizability of the results. The participants were middle-aged and older (age≥40 years), so these results may not apply in younger adults. However, mortality events are not common in individuals less than 40 years of age. We only assessed anthropometric indicators twice in an interval of about 4 to 5 years, the potential for misclassification cannot be excluded. In addition, residual and unmeasured confounding (eg, lifestyle changes and undiagnosed chronic illness) could not be completely ruled out. Lastly, we did not adjust menopausal status in women in the DFTJ cohort given that nearly all women (99.7%) were postmenopausal. We did not adjust menopausal status in women in the Kailuan study, given only a small proportion of women (9.5%) provided information of menopausal status.

## Conclusions

This study found U-shape associations of changes in weight and waist circumference with all-cause mortality in middle-aged and older Chinese adults. Notably, we found that weight loss with concurrent waist circumference gain was associated with a higher mortality risk. These findings provide insights into understanding the complex associations between changes in anthropometric measures and mortality and underscore the importance of maintaining body weight and waist circumference in middle-aged and older populations.

## Notes

Supplement. **eAppendix.** Supplementary Methods **eFigure 1.** Flow Chart of Participant Recruitment From the DFTJ Cohort and Kailuan Study **eFigure 2.** The Restricted Cubic Spline for the Association Between the Changes in Weight and All-Cause Mortality in the DFTJ Cohort and Kailuan Study **eFigure 3.** The Restricted Cubic Spline for the Association Between the Changes in Waist Circumference and All-Cause Mortality in the DFTJ Cohort and Kailuan Study **eFigure 4.** Adjusted Hazard Ratios for All-Cause Mortality Based on the Joint Changes in Weight and Waist Circumference in DFTJ Cohort and Kailuan Study **eFigure 5.** Associations Between Weight Change Categories and All-Cause Mortality, Stratified by Age, Sex, BMI, Waist Circumference, Diabetes, and Physical Activity at Baseline **eFigure 6.** Associations Between Waist Circumference Change Categories and All-Cause Mortality, Stratified by Age, Sex, BMI, Waist Circumference, Diabetes, and Physical Activity at Baseline **eFigure 7.** Adjusted Hazard Ratios for All-Cause Mortality Based on the Joint Changes in Weight and Waist Circumference (Random-effects Meta-analyses) **eFigure 8.** Adjusted Hazard Ratios for All-Cause Mortality Based on the Joint Changes in Weight and Waist Circumference (Excluding 652 Deaths Within Two Years, n=57 480) **eFigure 9.** Adjusted Hazard Ratios for All-Cause Mortality Stratified by Age Based on the Joint Changes in Weight and Waist Circumference **eFigure 10.** Adjusted Hazard Ratios for All-Cause Mortality Stratified by Sex Based on the Joint Changes in Weight and Waist Circumference **eFigure 11.** Adjusted Hazard Ratios for All-Cause Mortality Stratified by Baseline BMI Based on the Joint Changes in Weight and Waist Circumference **eFigure 12.** Adjusted Hazard Ratios for All-Cause Mortality Stratified by Baseline Waist Circumference Based on the Joint Changes in Weight and Waist Circumference **eFigure 13.** Adjusted Hazard Ratios for All-Cause Mortality Stratified by Physical Activity Level Based on the Joint Changes in Weight and Waist Circumference **eFigure 14.** Adjusted Hazard Ratios for All-Cause Mortality Among Never Smokers Based on the Joint Changes in Weight and Waist Circumference (n=38 340) **eFigure 15.** Adjusted Hazard Ratios for All-Cause Mortality Based on the Joint Changes in Weight and Waist Circumference (Participants With BMI<28 and Without Diabetes, n=42 281) **eFigure 16.** Adjusted Hazard Ratios for All-Cause Mortality Stratified by Diabetes Based on the Joint Changes in Weight and Waist Circumference **eTable 1.** Other Variables in the Associations Between Weight Change Categories and All-Cause Mortality **eTable 2.** Other Variables in the Associations Between Waist Circumference Change Categories and All-Cause Mortality **eTable 3.** Basic Characteristics of the Participants by the Joint Categories of Weight and Waist Circumference Change **eTable 4.** Associations Between Weight Change Categories and All-Cause Mortality Among Never Smokers **eTable 5.** Associations Between Waist Circumference Change Categories and All-Cause Mortality Among Never Smokers **eTable 6.** Associations Between Weight Change Categories and All-Cause Mortality (Random-effects Meta-analyses) **eTable 7.** Associations Between Waist Circumference Change Categories and All-Cause Mortality (Random-effects Meta-analyses) **eTable 8.** Associations of Baseline Characteristics With Weight Change and Waist Circumference Change **eTable 9.** Associations of Baseline Characteristics With Joint Changes in Weight and Waist Circumference
eReferences
