# Supplementary material for: Analysis of Changes in Weight, Waist Circumference, or Both, and All-Cause Mortality in Chinese Adults
Source: JAMA Netw Open. 2022 Aug 8;5(8):e2225876. doi: 10.1001/jamanetworkopen.2022.25876 (PMC9361078; doi:10.1001/jamanetworkopen.2022.25876)
Supplement: Supplement. — eAppendix. Supplementary Methods eFigure 1. Flow Chart of Participant Recruitment From the DFTJ Cohort and Kailuan Study eFigure 2. The Restricted Cubic Spline for the Association Between the Changes in Weight and All-Cause Mortality in the DFTJ Cohort and Kailuan Study eFigure 3. The Restricted Cubic Spline for the Association Between the Changes in Waist Circumference and All-Cause Mortality in the DFTJ Cohort and Kailuan Study eFigure 4. Adjusted Hazard Ratios for All-Cause Mortality Based on the Joint Changes in Weight and Waist Circumference in DFTJ Cohort and Kailuan Study eFigure 5. Associations Between Weight Change Categories and All-Cause Mortality, Stratified by Age, Sex, BMI, Waist Circumference, Diabetes, and Physical Activity at Baseline eFigure 6. Associations Between Waist Circumference Change Categories and All-Cause Mortality, Stratified by Age, Sex, BMI, Waist Circumference, Diabetes, and Physical Activity at Baseline eFigure 7. Adjusted Hazard Ratios for All-Cause Mortality Based on the Joint Changes in Weight and Waist Circumference (Random-effects Meta-analyses) eFigure 8. Adjusted Hazard Ratios for All-Cause Mortality Based on the Joint Changes in Weight and Waist Circumference (Excluding 652 Deaths Within Two Years, n=57 480) eFigure 9. Adjusted Hazard Ratios for All-Cause Mortality Stratified by Age Based on the Joint Changes in Weight and Waist Circumference eFigure 10. Adjusted Hazard Ratios for All-Cause Mortality Stratified by Sex Based on the Joint Changes in Weight and Waist Circumference eFigure 11. Adjusted Hazard Ratios for All-Cause Mortality Stratified by Baseline BMI Based on the Joint Changes in Weight and Waist Circumference eFigure 12. Adjusted Hazard Ratios for All-Cause Mortality Stratified by Baseline Waist Circumference Based on the Joint Changes in Weight and Waist Circumference eFigure 13. Adjusted Hazard Ratios for All-Cause Mortality Stratified by Physical Activity Level Based on the Joint Changes in Weight and Waist C [file jamanetwopen-e2225876-s001.pdf]

Supplemental Online Content

Yuan Y, Liu K, Zheng M, et al. Analysis of changes in weight, waist circumference, or both, and all-cause mortality in Chinese adults. *JAMA Netw Open.* 2022;5(8):e2225876. doi:10.1001/jamanetworkopen.2022.25876

eAppendix. Supplementary Methods

eFigure 1. Flow Chart of Participant Recruitment From the DFTJ Cohort and Kailuan Study

eFigure 2. The Restricted Cubic Spline for the Association Between the Changes in Weight and All-Cause Mortality in the DFTJ Cohort and Kailuan Study

eFigure 3 . The Restricted Cubic Spline for the Association Between the Changes in Waist Circumference and All-Cause Mortality in the DFTJ Cohort and Kailuan Study

eFigure 4. Adjusted Hazard Ratios for All-Cause Mortality Based on the Joint Changes in Weight and Waist Circumference in DFTJ Cohort and Kailuan Study

eFigure 5. Associations Between Weight Change Categories and All-Cause Mortality, Stratified by Age, Sex, BMI, Waist Circumference, Diabetes, and Physical Activity at Baseline

eFigure 6. Associations Between Waist Circumference Change Categories and All-Cause Mortality, Stratified by Age, Sex, BMI, Waist Circumference, Diabetes, and Physical Activity at Baseline

eFigure 7. Adjusted Hazard Ratios for All-Cause Mortality Based on the Joint Changes in Weight and Waist Circumference (Random-effects Meta-analyses)

© 2022 Yuan Y et al. *JAMA Network Open.*

eFigure 8. Adjusted Hazard Ratios for All-Cause Mortality Based on the Joint Changes in Weight and Waist Circumference (Excluding 652 Deaths Within Two Years, n=57 480)

eFigure 9. Adjusted Hazard Ratios for All-Cause Mortality Stratified by Age Based on the Joint Changes in Weight and Waist Circumference

eFigure 10. Adjusted Hazard Ratios for All-Cause Mortality Stratified by Sex Based on the Joint Changes in Weight and Waist Circumference

eFigure 11. Adjusted Hazard Ratios for All-Cause Mortality Stratified by Baseline BMI Based on the Joint Changes in Weight and Waist Circumference

eFigure 12. Adjusted Hazard Ratios for All-Cause Mortality Stratified by Baseline Waist Circumference Based on the Joint Changes in Weight and Waist Circumference

eFigure 13. Adjusted Hazard Ratios for All-Cause Mortality Stratified by Physical Activity Level Based on the Joint Changes in Weight and Waist Circumference

eFigure 14. Adjusted Hazard Ratios for All-Cause Mortality Among Never Smokers Based on the Joint Changes in Weight and Waist Circumference (n=38 340).

eFigure 15. Adjusted Hazard Ratios for All-Cause Mortality Based on the Joint Changes in Weight and Waist Circumference (Participants With BMI<28 and Without Diabetes, n=42 281)

eFigure 16. Adjusted Hazard Ratios for All-Cause Mortality Stratified by Diabetes Based on the Joint Changes in Weight and Waist Circumference

eTable 1. Other Variables in the Associations Between Weight Change Categories and All-Cause Mortality

eTable 2. Other Variables in the Associations Between Waist Circumference Change Categories and All-Cause Mortality

eTable 3. Basic Characteristics of the Participants by the Joint Categories of Weight and Waist Circumference Change

eTable 4. Associations Between Weight Change Categories and All-Cause Mortality Among Never Smokers.

eTable 5. Associations Between Waist Circumference Change Categories and All-Cause Mortality Among Never Smokers

© 2022 Yuan Y et al. *JAMA Network Open.*

eTable 6. Associations Between Weight Change Categories and All-Cause Mortality (Random-effects Meta-analyses)
eTable 7. Associations Between Waist Circumference Change Categories and All-Cause Mortality (Random-effects Meta-analyses)
eTable 8. Associations of Baseline Characteristics With Weight Change and Waist Circumference Change
eTable 9. Associations of Baseline Characteristics With Joint Changes in Weight and Waist Circumference
eReferences

This supplemental material has been provided by the authors to give readers additional information about their work.

eAppendix. Supplementary Methods

Assessment of covariates

In the DFTJ cohort, weekly hours of physical activity were calculated by multiplying the frequency of each week by the duration of each exercise session. The dietary intake was categorized to three items according to previous literatures,[1,2] including daily consumption of fruit and vegetables and red meat 1 to 6 days per week (favorable pattern), less than daily consumption of fruit and vegetables but daily consumption of meat (unfavorable pattern), and intermediate pattern. In the Kailuan study, physical activity was classified as regular (more than 20 minutes per time, three times per week), occasional (more than 20 minutes per time, one to two times per week), and no physical activity.[3] Given the lack of detailed dietary data during 2006 to 2010, total salt intake was used as a surrogate measure for diet quality,[3] which was classified as <6 gram per day (favorable pattern), 6 to 9 g/day (intermediate pattern), and ≥10 g/day (unfavorable pattern). Blood pressure was measured on the left upper arm with the participants in the seated position after a brief rest. In both cohorts, hypertension was defined as measured systolic blood pressure ≥140 mmHg or diastolic blood pressure ≥90 mmHg, or self-reported diagnosis of hypertension, or use of antihypertensive medications. Fasting blood glucose was measured with the Architect Ci8200 analyzer (Abbott Laboratories, Abbott Park, Illinois, USA) in DFTJ cohort, and the Hitachi 747 autoanalyzer (Hitachi, Tokyo, Japan) in Kailuan study. Diabetes was defined as fasting glucose level ≥7.0 mmol/L, self-reported diagnosis of diabetes, or use of antidiabetic medications (insulin or oral hypoglycemic agents) in both cohorts.

Statistical analysis

Person-years for each participant were calculated from the date of follow-up visit (2013 in the DFTJ cohort, and 2010-2011 in Kailuan study) until the date of death, or the end of follow-up (December 31, 2018), whichever came first. To minimize sample size reduction due to missing covariates (0.2%-1.0% for DFTJ cohort and 0.1%-0.2% for Kailuan study), indicator variables were created for missing categorical variables. We utilized Poisson regression models with robust standard errors to estimate incident rates and incident rate ratio (IRRs) and 95% CIs of mortality, and adjusted for age and sex.[4] The absolute rate difference (ARD) was estimated by $I \times (IRR - 1)$ (I indicates the incidence among the reference category).[4]

To examine the associations of the changes in weight or waist with all-cause mortality, we fitted restricted-cubic-spline regression with three knots (5th, 50th, 95th) in the two cohorts separately. Based on the distributions of weight change and waist change in each cohort, we replaced

© 2022 Yuan Y et al. *JAMA Network Open.*

all observations above the 99.5 or below the 0.5 percentiles with the 99.5 or 0.5 percentiles, respectively, to minimize the influence of outliers in the splines. Multivariate models were stratified by age at risk (5-year interval),[5] sex, and adjusted for height, smoking status (never, previous, or current smoker), alcohol consumption (never, previous, or current drinker), dietary pattern (favorable, intermediate, and unfavorable patterns), educational attainment (primary school or below, middle school, high school or beyond), and physical activity (hours per week in DFTJ, and no or occasional or regular physical activity in Kailuan study), diabetes status (yes or no), and hypertension status (yes or no). In stratified analyses, we further examined the associations of changes in weight or waist with the all-cause mortality among prespecified baseline subgroups based on age (<60 years, ≥60 years), sex (male, female), BMI at recruitment (<24 kg/m^2, ≥24 kg/m^2), waist circumference (<85 cm for male or < 80 cm for female, ≥85 cm for male or ≥80 cm for female), diabetes status at recruitment, or physical activity level (low or high). In the DFTJ cohort, low physical activity was defined as ≤5 h/week, while high physical activity was defined as >5 h/week. In the Kailuan study, low physical activity was defined as no physical activity, while high physical activity was defined as occasional and regular physical activity. The percentages of low physical activity in both cohorts were similar (30.3% in DFTJ and 30.1% in Kailuan). To account for the possibility of residual confounding by smoking,[6] we conducted sensitivity analyses by restricting to never smokers. To further reduce the potential for reverse causality, we performed analyses excluding cases events occurred within the first two years after the follow-up visit (n = 652).

To explore the potential risk factors of the weight and waist change, we used generalized linear regression model to evaluate the associations between baseline characteristics with the changes in weight/waist (continuous variable). Moreover, we applied multi-nominal logistic regression to explore the associations between basic characteristics with the joint changes in weight and waist circumference. The participants with stable weight or waist circumference were set as control group in the multinominal logistic regression. Different from the main analyses, we included all the covariates collected at baseline in the exploratory analyses.

© 2022 Yuan Y et al. *JAMA Network Open.*

eFigure 1. Flow Chart of Participant Recruitment From the DFTJ Cohort and Kailuan Study

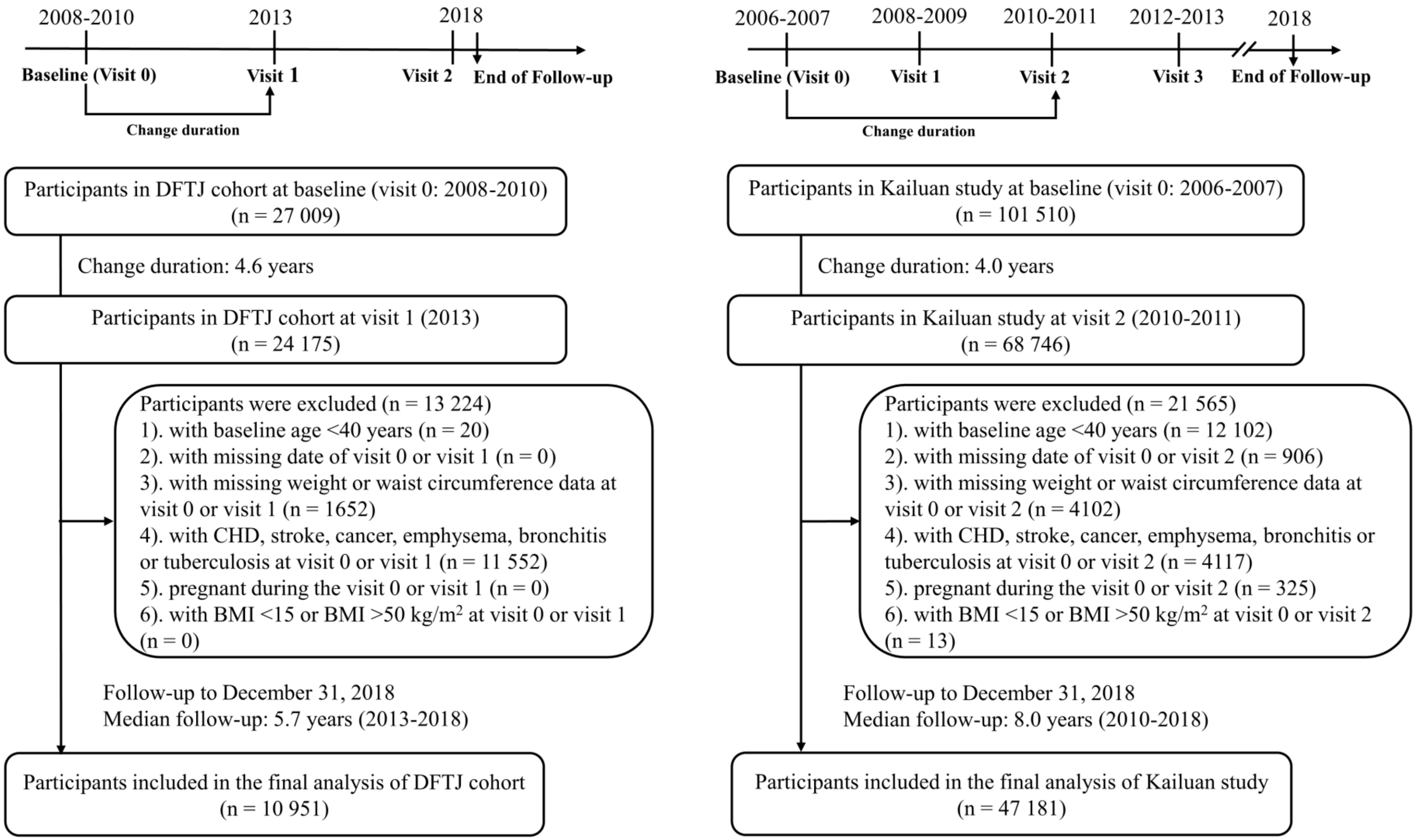

© 2022 Yuan Y et al. *JAMA Network Open.*

eFigure 2. The Restricted Cubic Spline for the Association Between the Changes in Weight and All-Cause Mortality in the DFTJ Cohort and Kailuan Study

Figure Legend: The curve represents adjusted HRs based on restricted cubic splines with knots at the 5th, 50th, and 95th percentiles of the distribution of weight change in each cohort (solid lines are HRs, dashed lines indicate 95% CIs), the reference values (HR=1) were set at where weight changes were equal to zero. The multivariable-adjusted model included weight change, height and weight at cohort recruitment, waist circumference change, smoking status, alcohol intake status, dietary pattern, educational attainment, physical activity, hypertension, and diabetes; and stratified by age at risk (5-year interval) and sex.

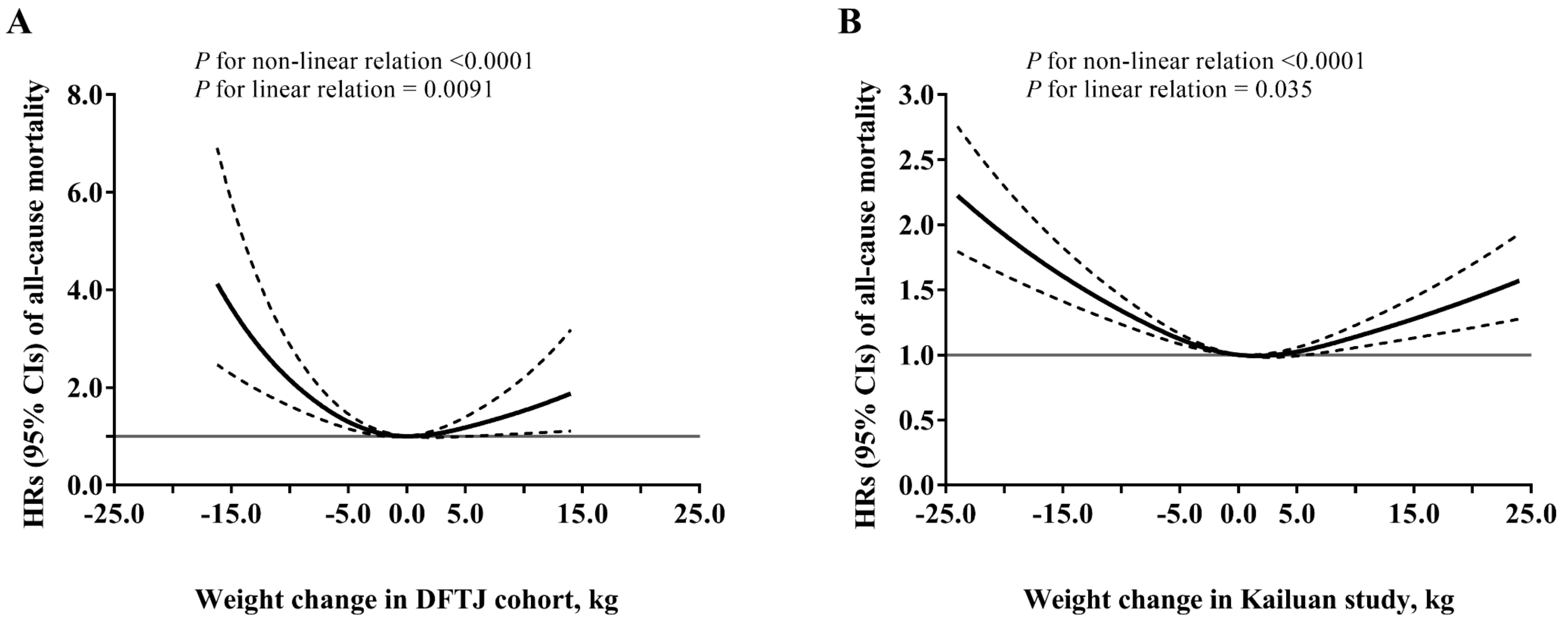

© 2022 Yuan Y et al. *JAMA Network Open.*

eFigure 3. The Restricted Cubic Spline for the Association Between the Changes in Waist Circumference and All-Cause Mortality in the DFTJ Cohort and Kailuan Study

Figure Legend: The curve represents adjusted HRs based on restricted cubic splines with knots at the 5th, 50th, and 95th percentiles of the distribution of waist circumference change in each cohort (solid lines are HRs, dashed lines indicate 95% CIs), the reference values (HR=1) were set at where waist circumference changes were equal to zero. The multivariable-adjusted model included waist circumference change, height and waist circumference at cohort recruitment, weight change, smoking status, alcohol intake status, dietary pattern, educational attainment, physical activity, hypertension, and diabetes; and stratified by age at risk (5-year interval) and sex.

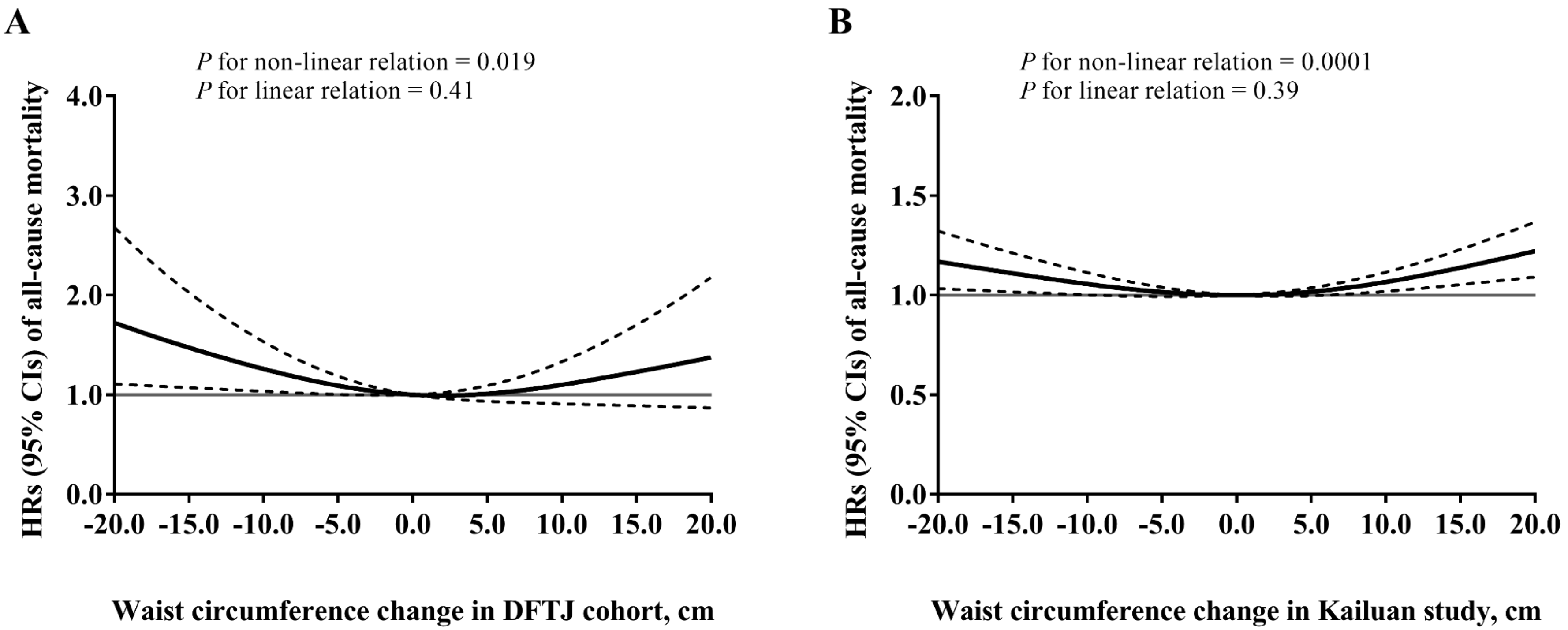

© 2022 Yuan Y et al. *JAMA Network Open.*

eFigure 4. Adjusted Hazard Ratios for All-Cause Mortality Based on the Joint Changes in Weight and Waist Circumference in DFTJ Cohort and Kailuan Study

Figure Legend: The multivariable-adjusted model included the combined categories of weight and waist circumference changes, weight, height and waist circumference at cohort recruitment, smoking status, alcohol intake status, dietary pattern, educational attainment, physical activity, hypertension, and diabetes; and stratified by age at risk (5-year interval) and sex. The majority of *P* for heterogeneity were larger than 0.05. Only one group were statistically significant between two studies (*P* for heterogeneity=0.016 for the weight stable and waist loss group).

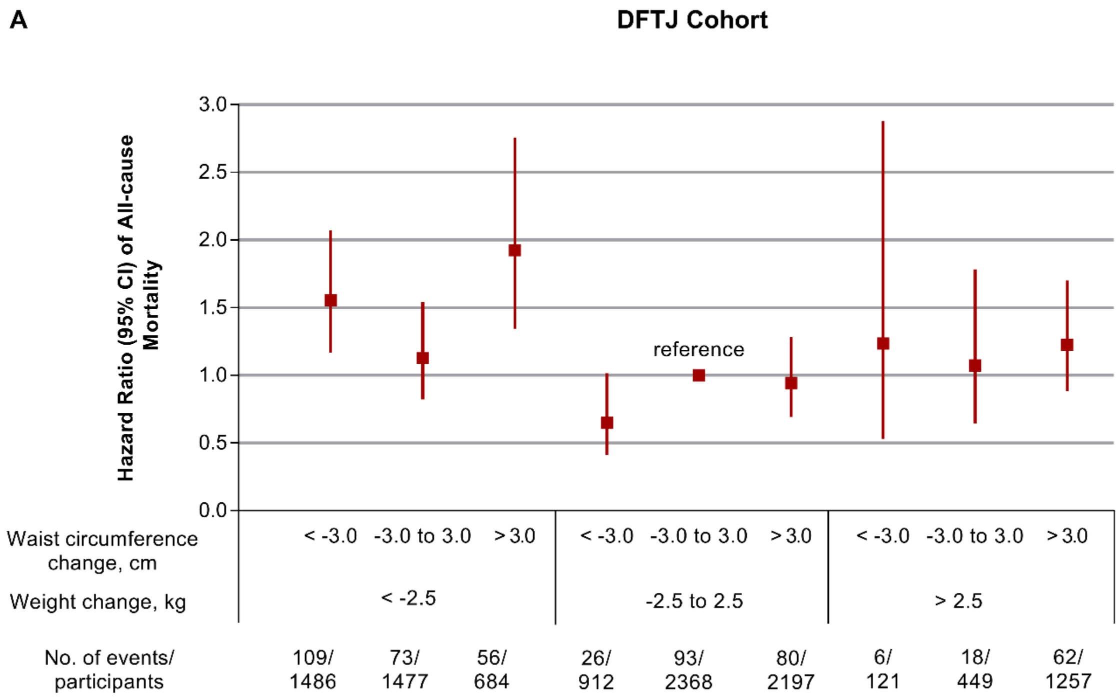

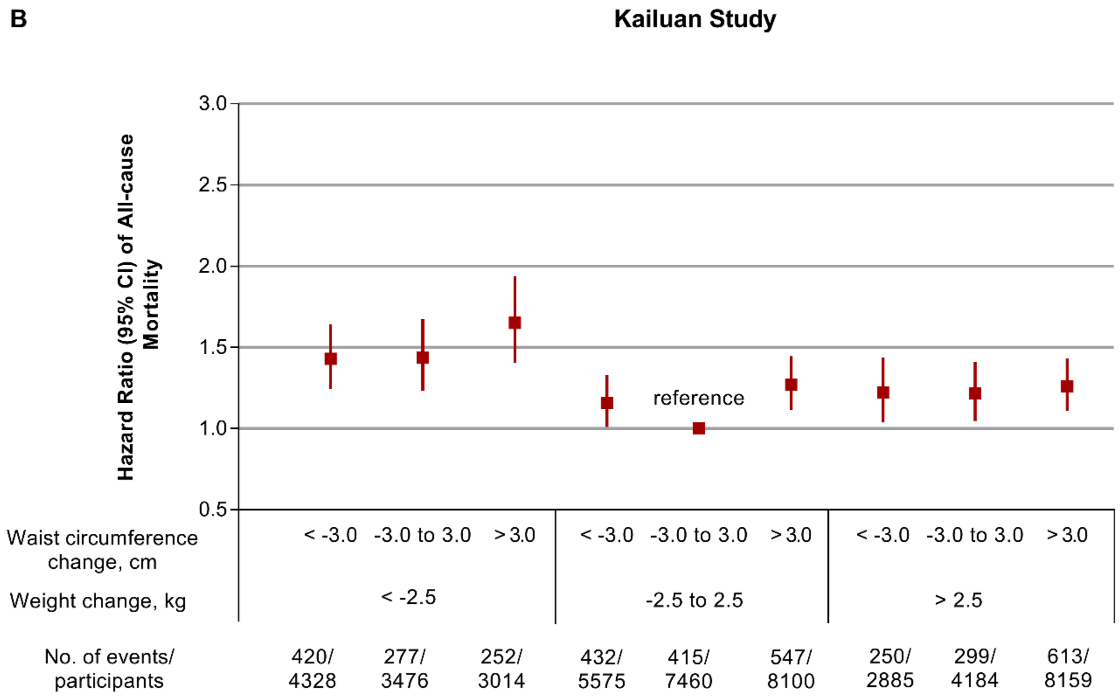

© 2022 Yuan Y et al. *JAMA Network Open.*

eFigure 5. Associations Between Weight Change Categories and All-Cause Mortality, Stratified by Age, Sex, BMI, Waist Circumference, Diabetes, and Physical Activity at Baseline

Figure Legend: The multivariable models were adjusted for height and weight at cohort recruitment, waist circumference change (continuous variables), smoking status, alcohol intake status, dietary pattern, educational attainment, physical activity, hypertension, and diabetes (except for the strata variable), and stratified by age at risk (5-year interval) and sex. We conducted cohort-specific analyses, which were pooled together using fixed-effect meta-analyses. Weight change were classified into weight loss (lost >2.5 kg of weight), stable weight (change ±2.5 kg), and weight gain (gained >2.5 kg). Normal waist circumferences were defined as the waist circumference of male <85 cm or female <80 cm. High waist circumferences were defined as the waist circumference of male ≥85 cm or female ≥80cm. In the DFTJ cohort, low physical activity was defined as ≤5 h/week, while high physical activity was defined as >5 h/week. In the Kailuan study, low physical activity was defined as no physical activity, while high physical activity was defined as occasional and regular physical activity. The percentages of low physical activity in both cohorts were similar (30.3% in DFTJ and 30.1% in Kailuan).

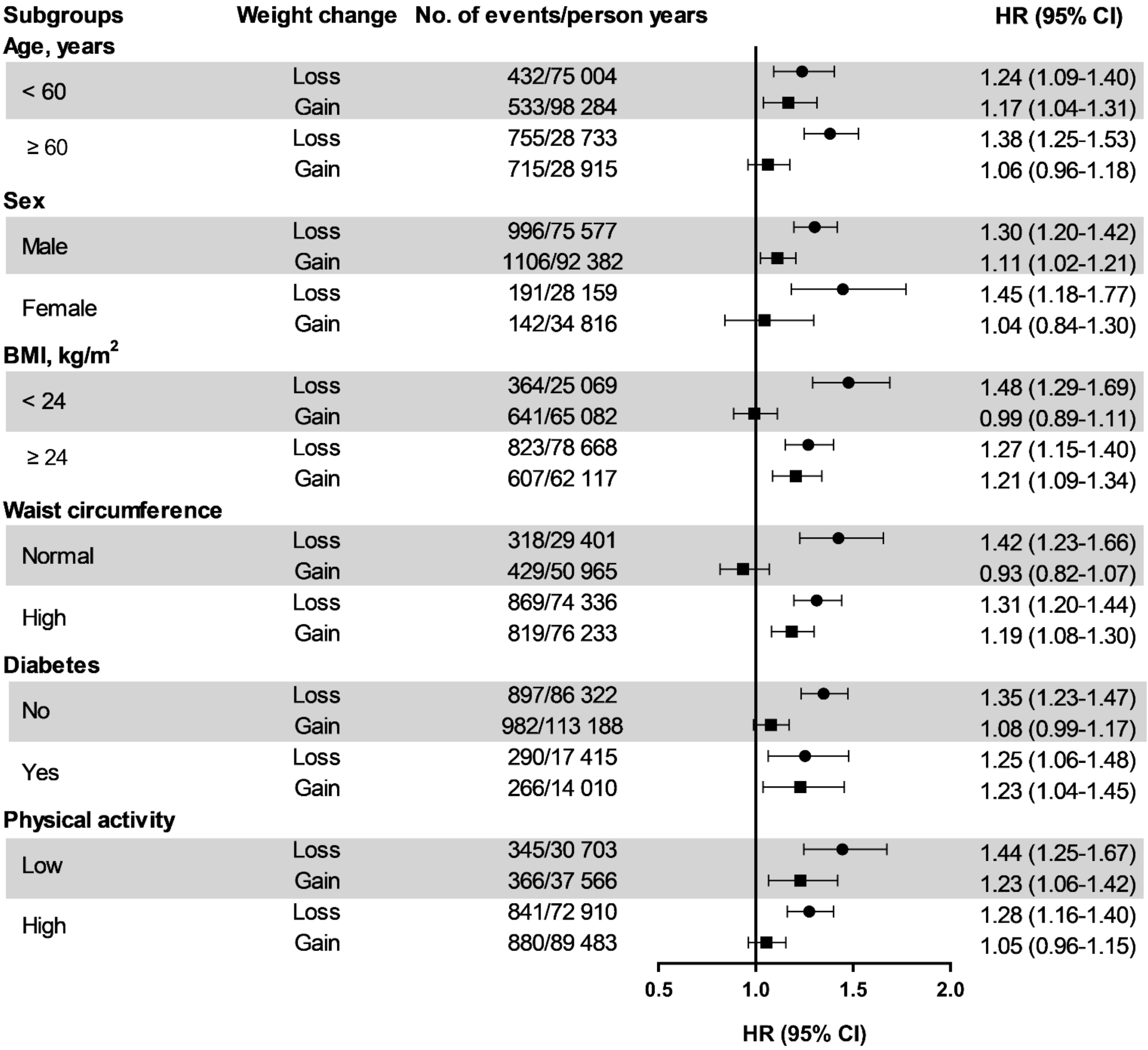

eFigure 6. Associations Between Waist Circumference Change Categories and All-Cause Mortality, Stratified by Age, Sex, BMI, Waist Circumference, Diabetes, and Physical Activity at Baseline

Figure Legend: The multivariable models were adjusted for height and waist circumference at cohort recruitment, weight change (continuous variables), smoking status, alcohol intake status, dietary pattern, educational attainment, physical activity, hypertension, and diabetes (except for the strata variable), and stratified by age at risk (5-year interval) and sex. We conducted cohort-specific analyses, which were pooled together using fixed-effect meta-analyses. Waist change were classified into waist loss (lost >3 cm of waist), stable waist (waist change ±3 cm), and waist gain (gained >3 cm). Normal waist circumferences were defined as the waist circumference of male <85 cm or female <80 cm. High waist circumferences were defined as the waist circumference of male ≥85 cm or female ≥80cm. In the DFTJ cohort, low physical activity was defined as ≤5 h/week, while high physical activity was defined as >5 h/week. In the Kailuan study, low physical activity was defined as no physical activity, while high physical activity was defined as occasional and regular physical activity. The percentages of low physical activity in both cohorts were similar (30.3% in DFTJ and 30.1% in Kailuan).

Subgroups	Waist change	No. of events/person years	HR (95% CI)
**Age, years**			
< 60	Loss	474/82 434	1.16 (1.02-1.32)
	Gain	654/130 988	1.03 (0.92-1.17)
≥ 60	Loss	769/29 724	1.14 (1.02-1.27)
	Gain	956/41 196	1.17 (1.06-1.30)
**Sex**			
Male	Loss	1079/79 507	1.15 (1.05-1.26)
	Gain	1382/125 886	1.11 (1.02-1.20)
Female	Loss	164/32 651	1.06 (0.85-1.33)
	Gain	228/46 298	1.16 (0.95-1.43)
**BMI, kg/m^2**			
< 24	Loss	466/41 639	1.12 (0.98-1.29)
	Gain	724/70 585	1.08 (0.95-1.22)
≥ 24	Loss	777/70 519	1.08 (0.97-1.20)
	Gain	886/101 599	1.18 (1.07-1.31)
**Waist circumference**			
Normal	Loss	172/16 459	1.20 (1.00-1.45)
	Gain	749/88 309	1.07 (0.94-1.23)
High	Loss	1071/95 699	1.12 (1.02-1.23)
	Gain	861/83 875	1.13 (1.03-1.25)
**Diabetes**			
No	Loss	960/96 521	1.15 (1.04-1.26)
	Gain	1283/151 284	1.15 (1.05-1.25)
Yes	Loss	283/15 637	1.12 (0.94-1.33)
	Gain	327/20 900	1.02 (0.86-1.20)
**Physical activity**			
Low	Loss	363/32 562	1.19 (1.02-1.39)
	Gain	420/50 383	1.04 (0.90-1.21)
High	Loss	879/79 469	1.12 (1.02-1.24)
	Gain	1189/121 630	1.15 (1.05-1.26)

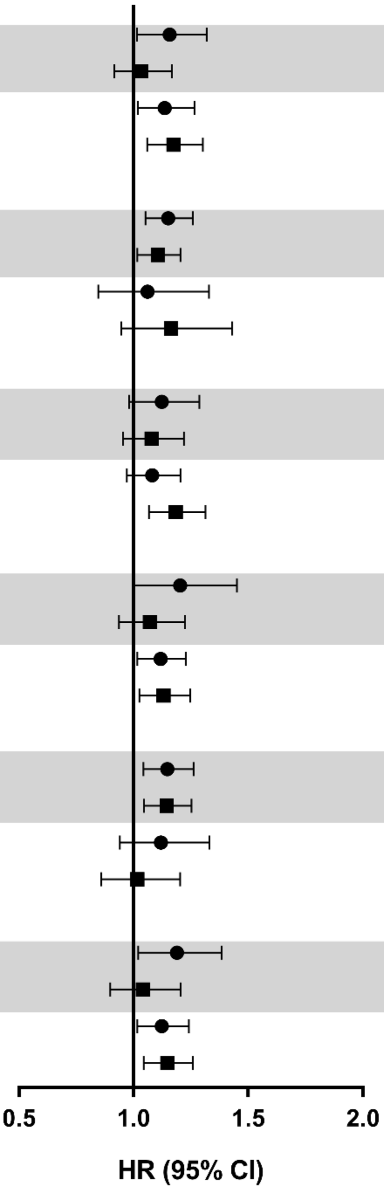

© 2022 Yuan Y et al. *JAMA Network Open.*

eFigure 7. Adjusted Hazard Ratios for All-Cause Mortality Based on the Joint Changes in Weight and Waist Circumference (Random-effects Meta-analyses).

Figure Legend: The multivariable-adjusted model included the joint categories of weight and waist circumference changes, weight, height and waist circumference at cohort recruitment, smoking status, alcohol intake status, dietary pattern, educational attainment, physical activity, hypertension, and diabetes; and stratified by age at risk (5-year interval) and sex. Cohort-specific results were pooled together using random-effects meta-analyses.

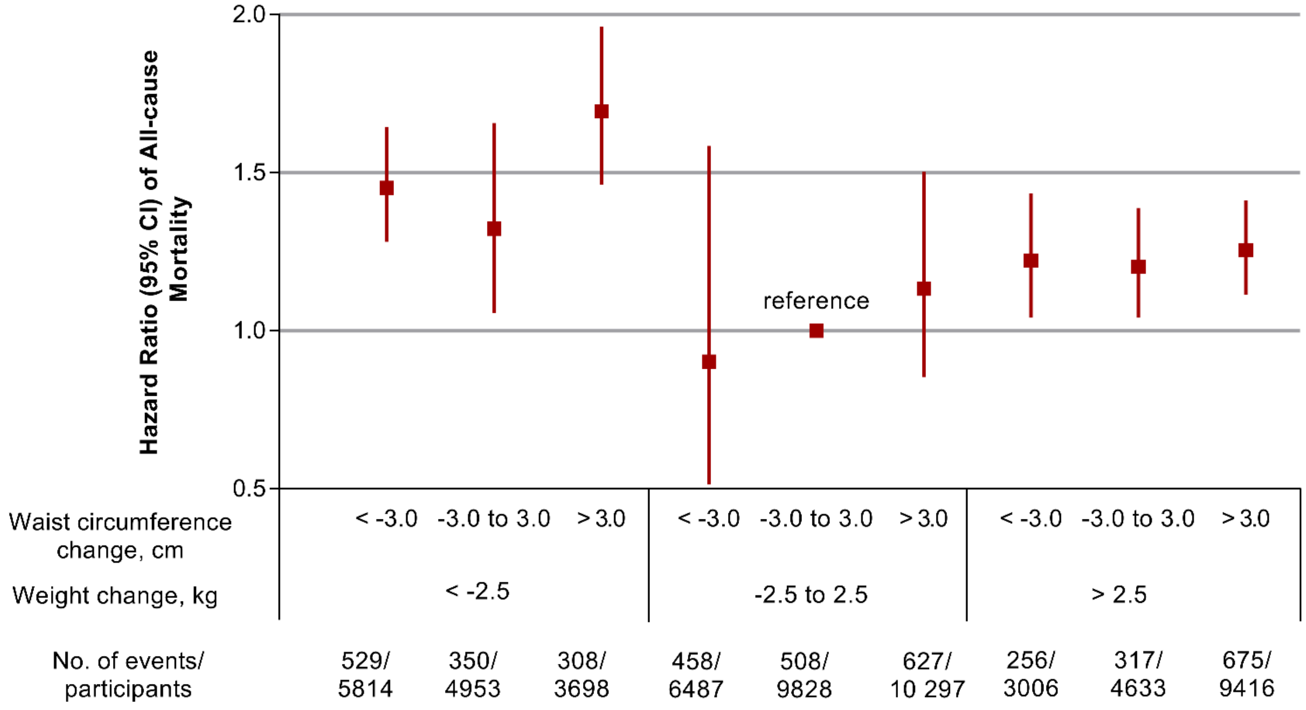

© 2022 Yuan Y et al. *JAMA Network Open.*

eFigure 8. Adjusted Hazard Ratios for All-Cause Mortality Based on the Joint Changes in Weight and Waist Circumference (Excluding 652 Deaths Within Two Years, n=57 480)

Figure Legend: The multivariable-adjusted model included the combined categories of weight and waist circumference changes, weight, height and waist circumference at cohort recruitment, alcohol intake status, dietary pattern, educational attainment, physical activity, hypertension, and diabetes; and stratified by age at risk (5-year interval) and sex. Cohort-specific results were pooled together using fixed-effect meta-analyses.

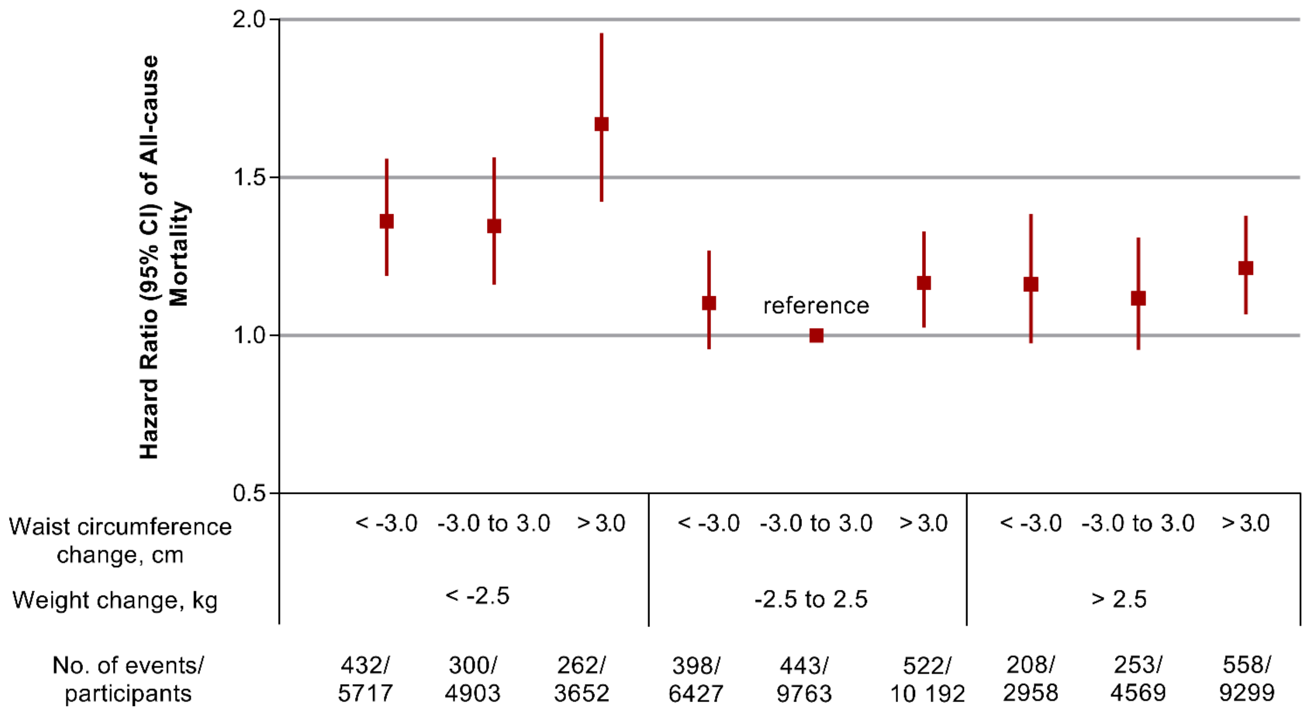

© 2022 Yuan Y et al. *JAMA Network Open.*

eFigure 9. Adjusted Hazard Ratios for All-Cause Mortality Stratified by Age Based on the Joint Changes in Weight and Waist Circumference

Figure Legend: The multivariable-adjusted model included the combined categories of weight and waist circumference changes, weight, height and waist circumference at cohort recruitment, alcohol intake status, dietary pattern, educational attainment, physical activity, hypertension, and diabetes; and stratified by age at risk (5-year interval) and sex. Cohort-specific results were pooled together using fixed-effect meta-analyses. Multiplicative *P* interaction for the age strata was 0.56 in the pooled meta-analyses.

A **Age <60 years**

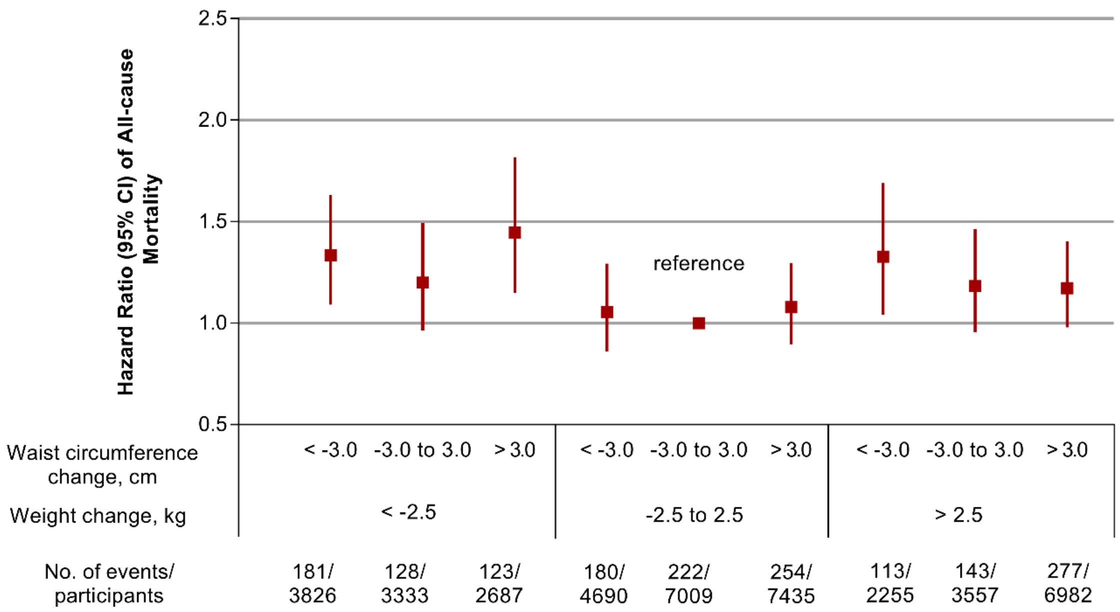

B **Age ≥60 years**

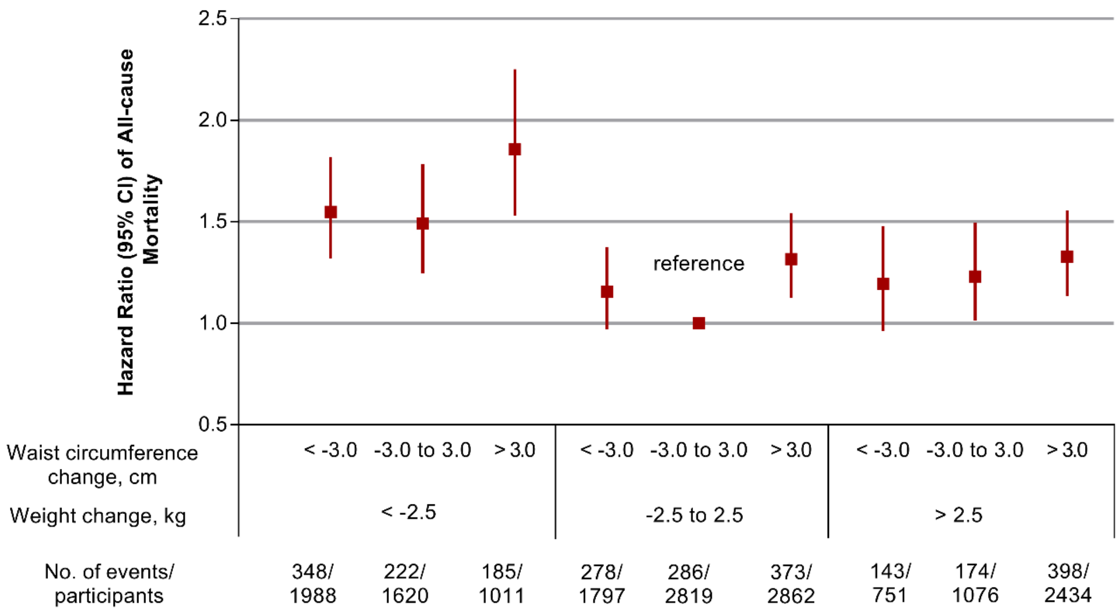

© 2022 Yuan Y et al. *JAMA Network Open.*

eFigure 10. Adjusted Hazard Ratios for All-Cause Mortality Stratified by Sex Based on the Joint Changes in Weight and Waist Circumference

Figure Legend: The multivariable-adjusted model included the combined categories of weight and waist circumference changes, weight, height and waist circumference at cohort recruitment, alcohol intake status, dietary pattern, educational attainment, physical activity, hypertension, and diabetes; and stratified by age at risk (5-year interval). Cohort-specific results were pooled together using fixed-effect meta-analyses. Multiplicative *P* interaction for the sex strata was 0.70 in the pooled meta-analyses.

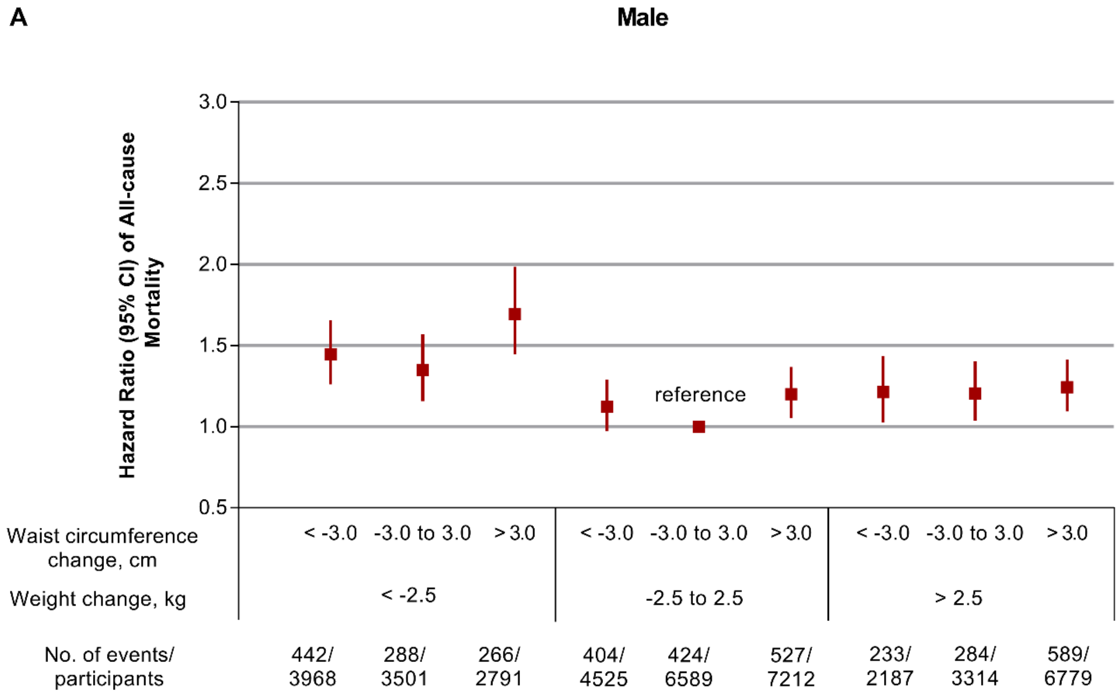

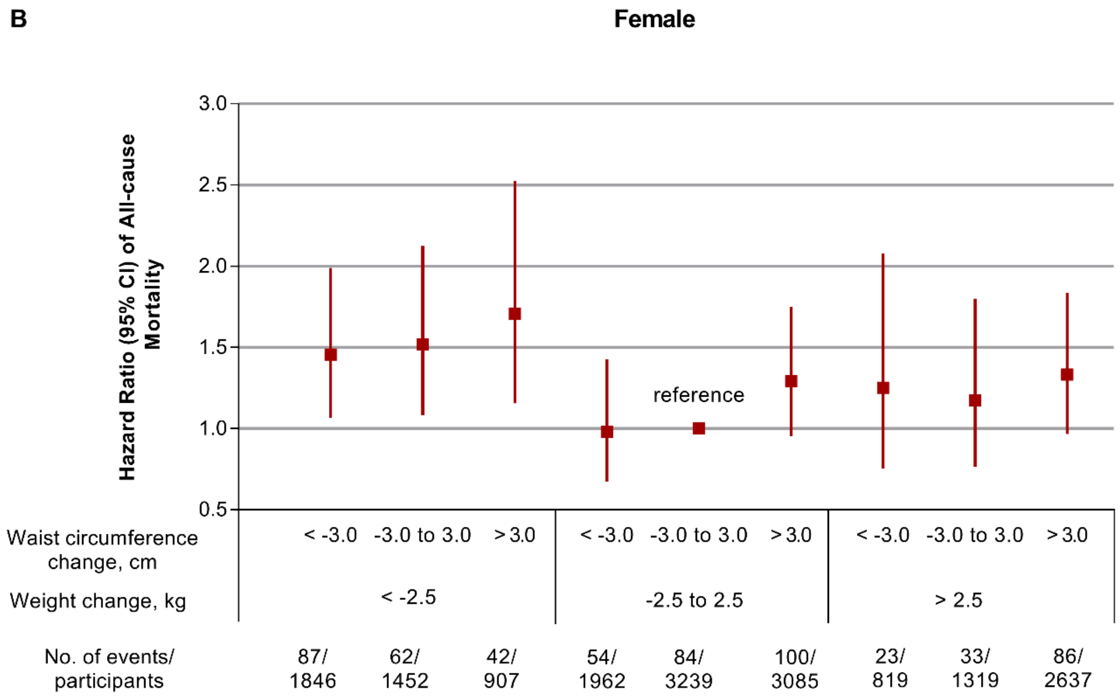

© 2022 Yuan Y et al. *JAMA Network Open.*

eFigure 11. Adjusted Hazard Ratios for All-Cause Mortality Stratified by Baseline BMI Based on the Joint Changes in Weight and Waist Circumference.

Figure Legend: The multivariable-adjusted model included the combined categories of weight and waist circumference changes, weight, height and waist circumference at cohort recruitment, alcohol intake status, dietary pattern, educational attainment, physical activity, hypertension, and diabetes; and stratified by age at risk (5-year interval) and sex. Cohort-specific results were pooled together using fixed-effect meta-analyses. Baseline BMI were categorized into <24 kg/m^2 and ≥24 kg/m^2. Multiplicative *P* interaction for the baseline BMI was 0.070 in the pooled meta-analyses.

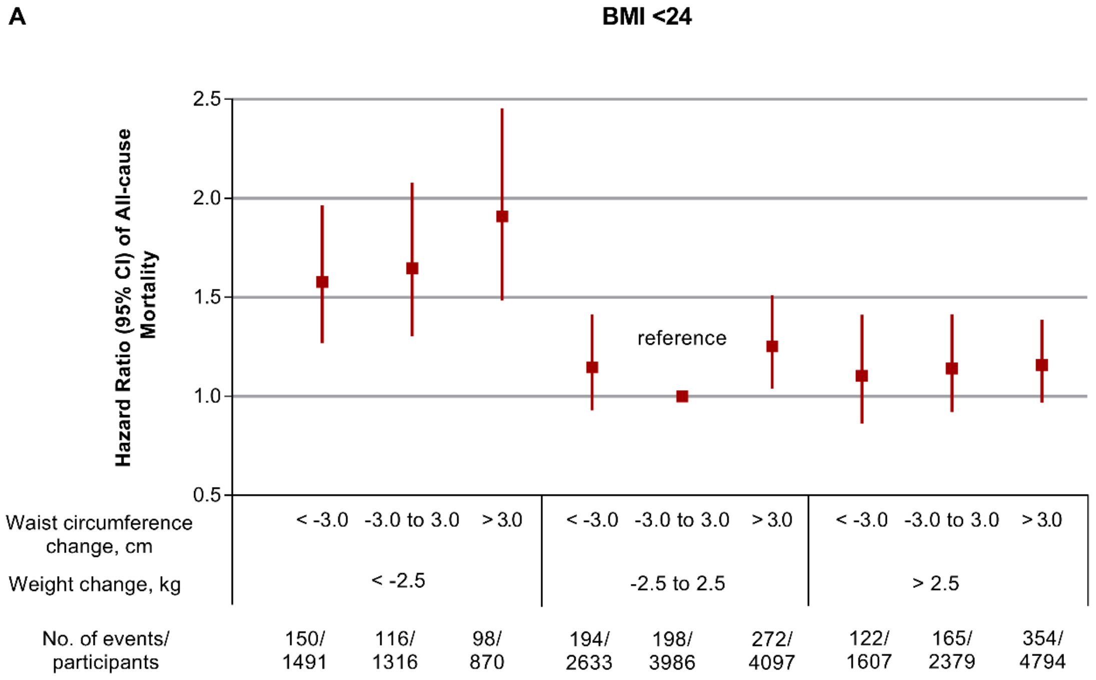

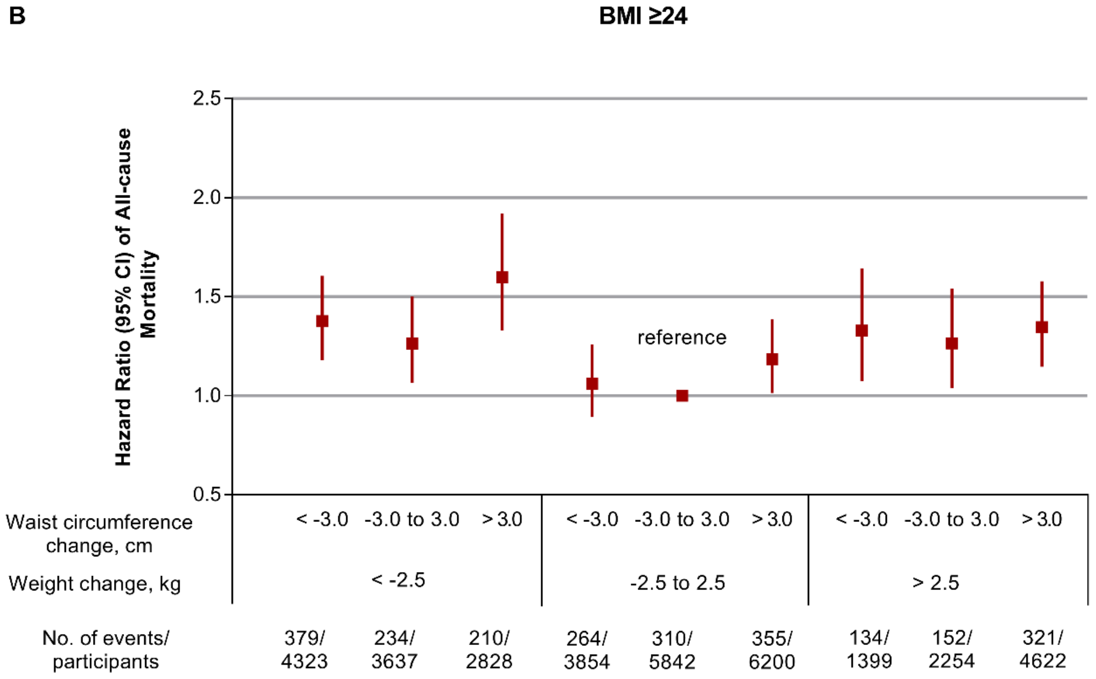

© 2022 Yuan Y et al. *JAMA Network Open.*

eFigure 12. Adjusted Hazard Ratios for All-Cause Mortality Stratified by Baseline Waist Circumference Based on the Joint Changes in Weight and Waist Circumference

Figure Legend: The multivariable-adjusted model included the combined categories of weight and waist circumference changes, weight, height and waist circumference at cohort recruitment, alcohol intake status, dietary pattern, educational attainment, physical activity, hypertension, and diabetes; and stratified by age at risk (5-year interval) and sex. Cohort-specific results were pooled together using fixed-effect meta-analyses. Normal waist circumferences were defined as the waist circumference of male <85 cm or female <80 cm. High waist circumferences were defined as the waist circumference of male ≥85 cm or female ≥80cm. Multiplicative *P* interaction for the baseline waist circumference was 0.013 in the pooled meta-analyses.

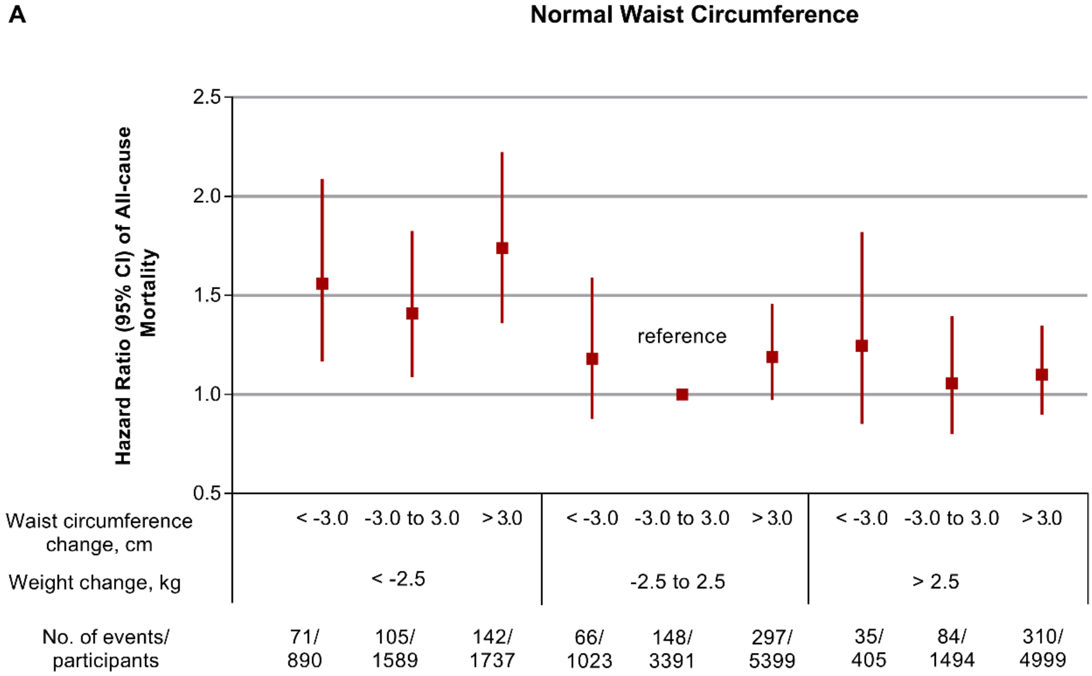

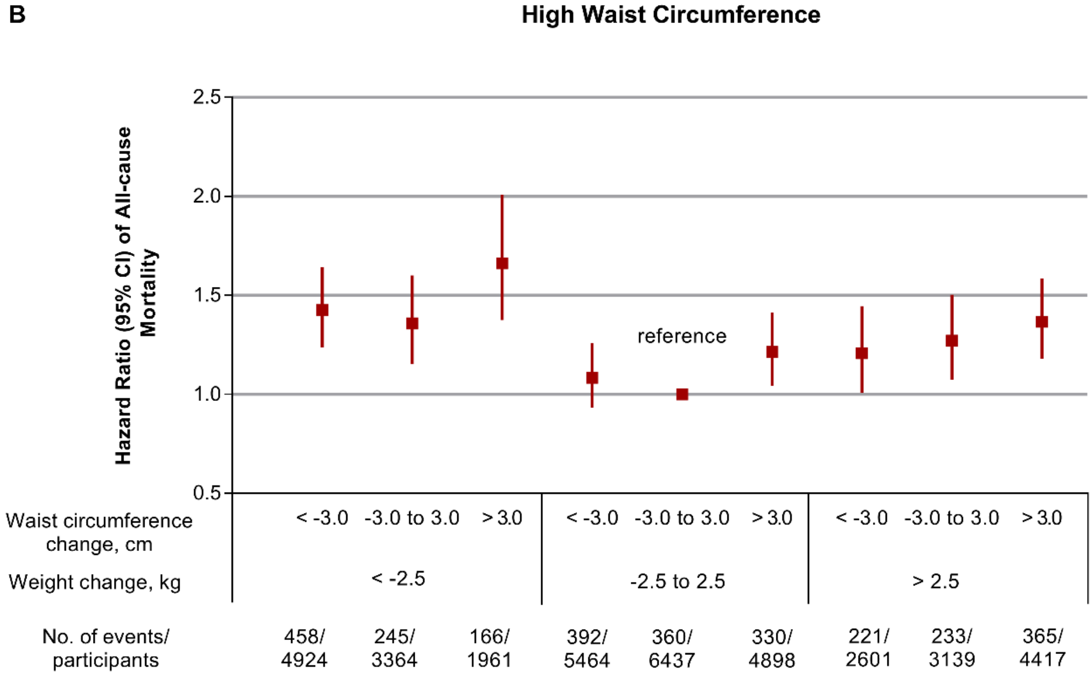

© 2022 Yuan Y et al. *JAMA Network Open.*

eFigure 13. Adjusted Hazard Ratios for All-Cause Mortality Stratified by Physical Activity Level Based on the Joint Changes in Weight and Waist Circumference

Figure Legend: The multivariable-adjusted model included the combined categories of weight and waist circumference changes, weight, height and waist circumference at cohort recruitment, alcohol intake status, dietary pattern, educational attainment, hypertension, and diabetes; and stratified by age at risk (5-year interval) and sex. Cohort-specific results were pooled together using fixed-effect meta-analyses. In the DFTJ cohort, low physical activity was defined as ≤5 h/week, while high physical activity was defined as >5 h/week. In the Kailuan study, low physical activity was defined as no physical activity, while high physical activity was defined as occasional and regular physical activity. The percentages of low physical activity in both cohorts were similar (30.3% in DFTJ and 30.1% in Kailuan). Multiplicative *P* interaction for the physical activity was 0.13 in the pooled meta-analyses.

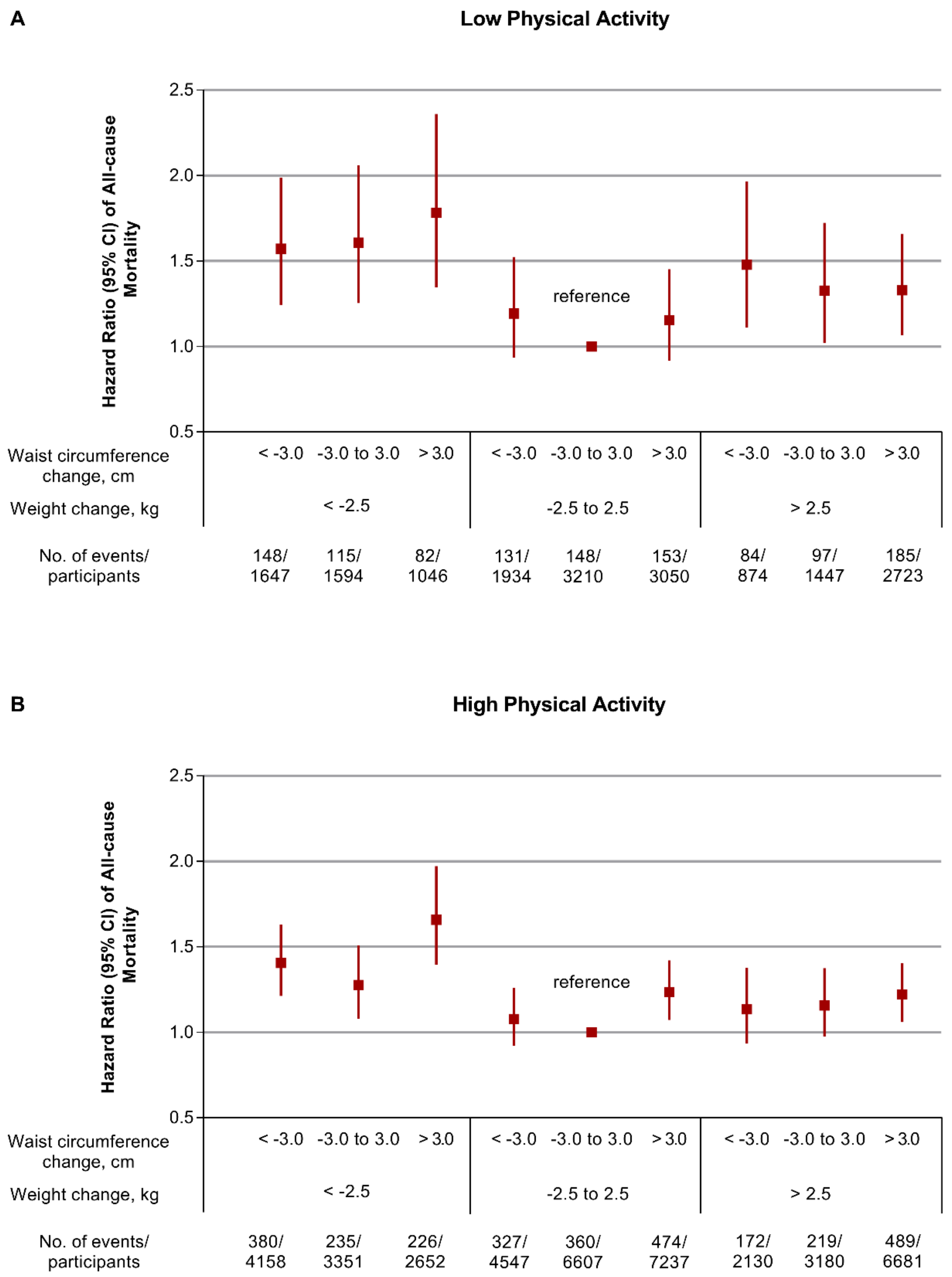

© 2022 Yuan Y et al. *JAMA Network Open.*

eFigure 14. Adjusted Hazard Ratios for All-Cause Mortality Among Never Smokers Based on the Joint Changes in Weight and Waist Circumference (n=38 340).

Figure Legend: The multivariable-adjusted model included the combined categories of weight and waist circumference changes, weight, height and waist circumference at cohort recruitment, alcohol intake status, dietary pattern, educational attainment, physical activity, hypertension, and diabetes; and stratified by age at risk (5-year interval) and sex. Cohort-specific results were pooled together using fixed-effect meta-analyses.

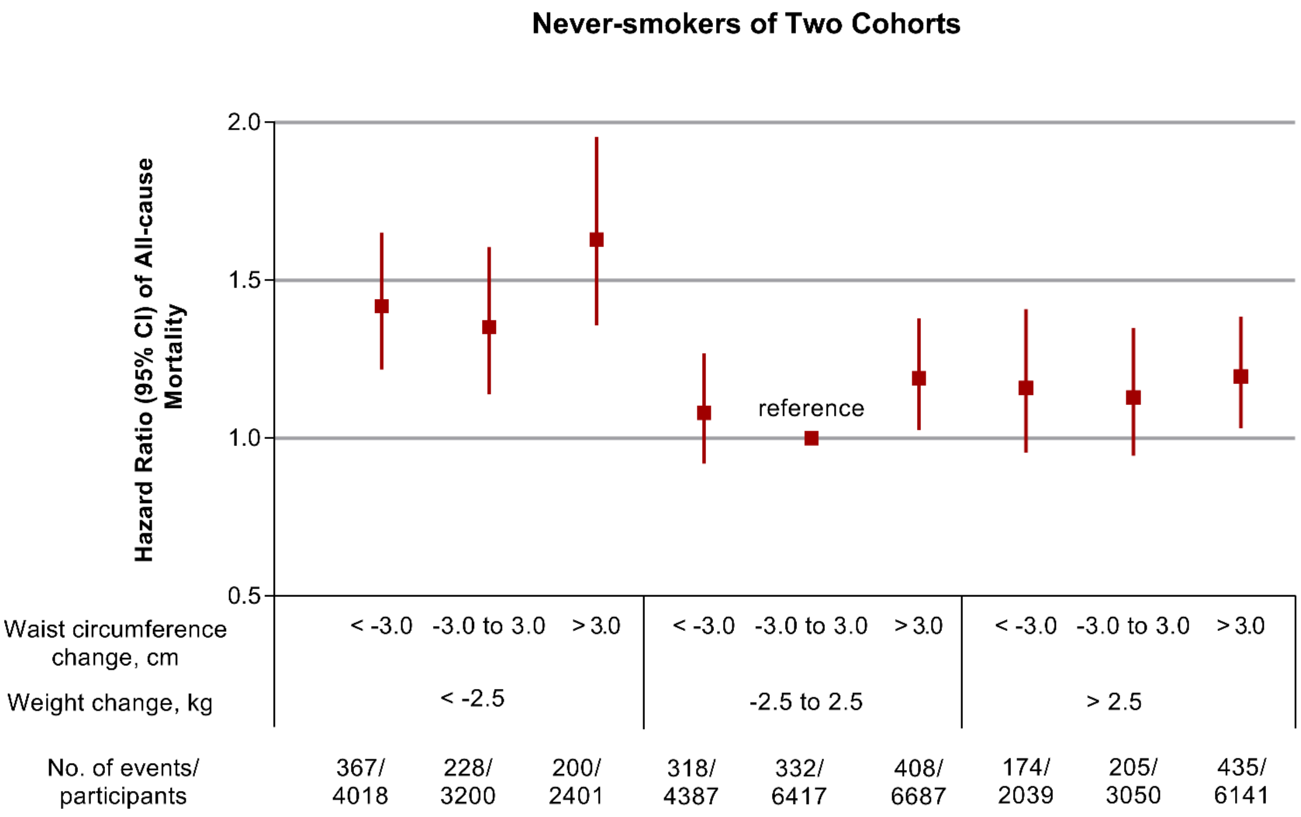

© 2022 Yuan Y et al. *JAMA Network Open.*

eFigure 15. Adjusted Hazard Ratios for All-Cause Mortality Based on the Joint Changes in Weight and Waist Circumference (Participants With BMI<28 and Without Diabetes, n=42 281).

Figure Legend: The multivariable-adjusted model included the combined categories of weight and waist circumference changes, weight, height and waist circumference at cohort recruitment, alcohol intake status, dietary pattern, educational attainment, physical activity, and hypertension; and stratified by age at risk (5-year interval) and sex. Cohort-specific results were pooled together using fixed-effect meta-analyses.

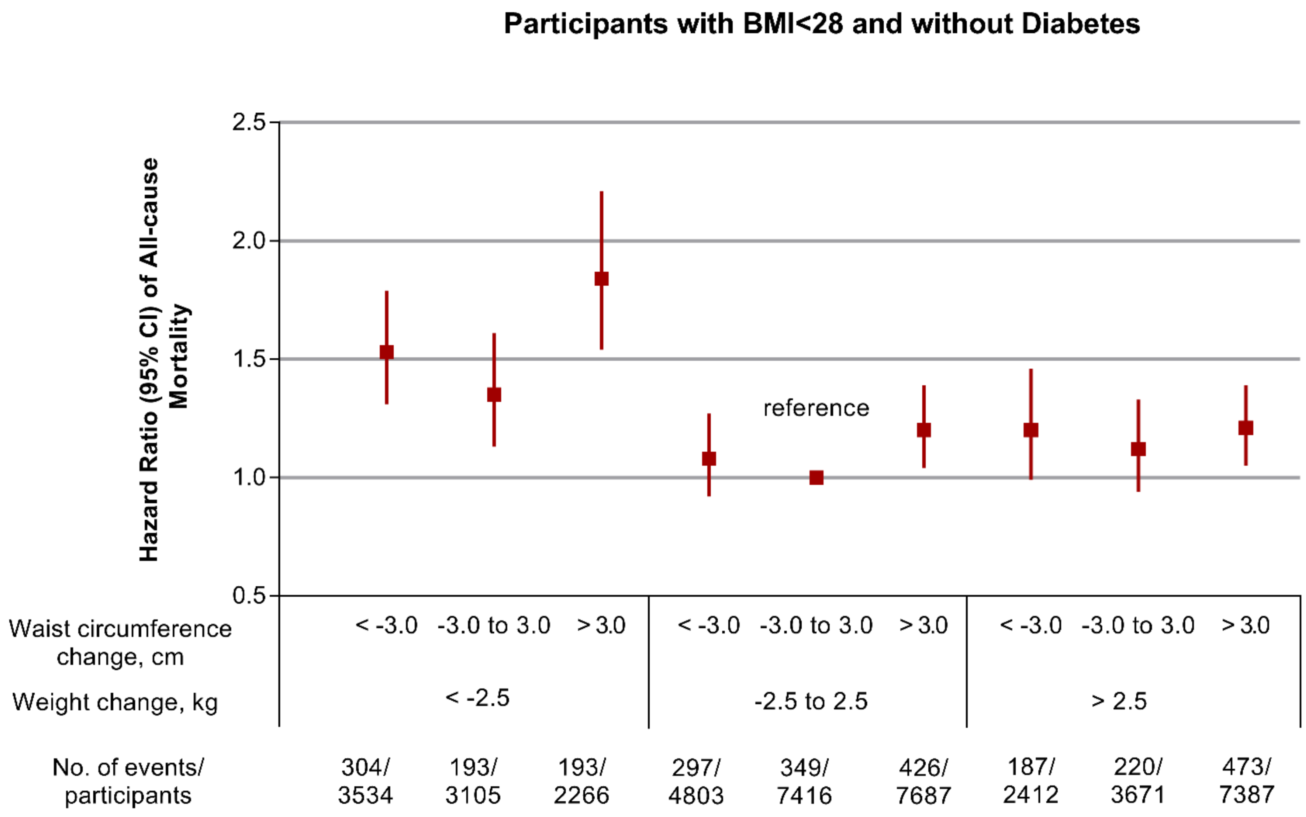

© 2022 Yuan Y et al. *JAMA Network Open.*

eFigure 16. Adjusted Hazard Ratios for All-Cause Mortality Stratified by Diabetes Based on the Joint Changes in Weight and Waist Circumference.

Figure Legend: The multivariable-adjusted model included the combined categories of weight and waist circumference changes, weight, height and waist circumference at cohort recruitment, alcohol intake status, dietary pattern, educational attainment, physical activity, and hypertension; and stratified by age at risk (5-year interval) and sex. Cohort-specific results were pooled together using fixed-effect meta-analyses. Multiplicative *P* interaction for the diabetes was 0.54 in the pooled meta-analyses.

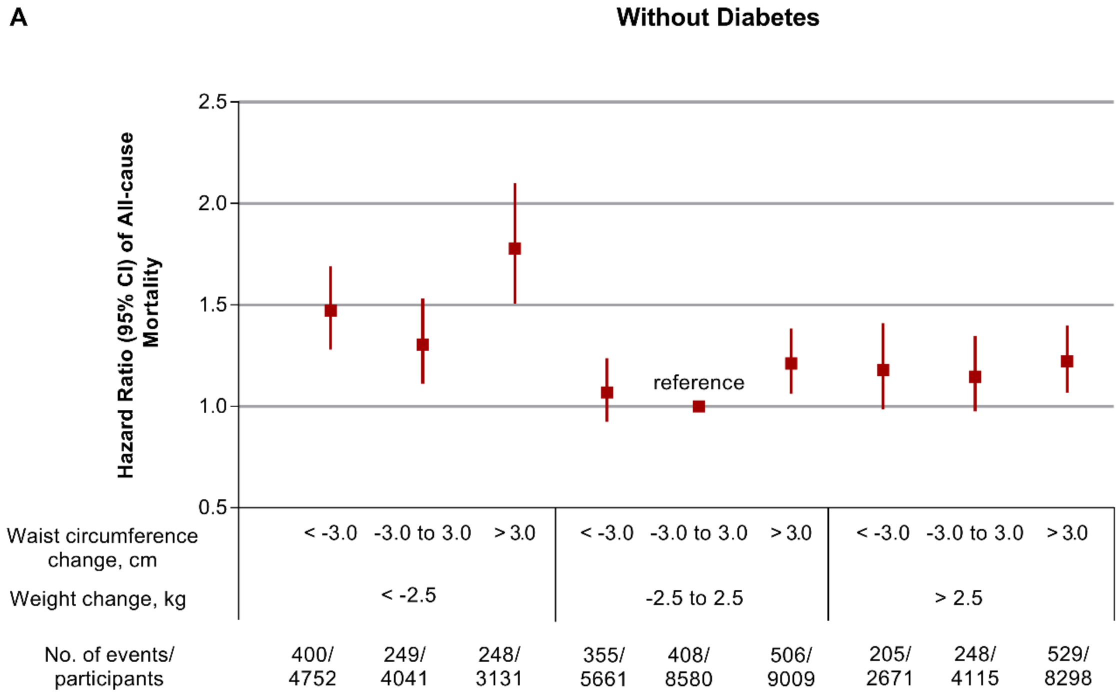

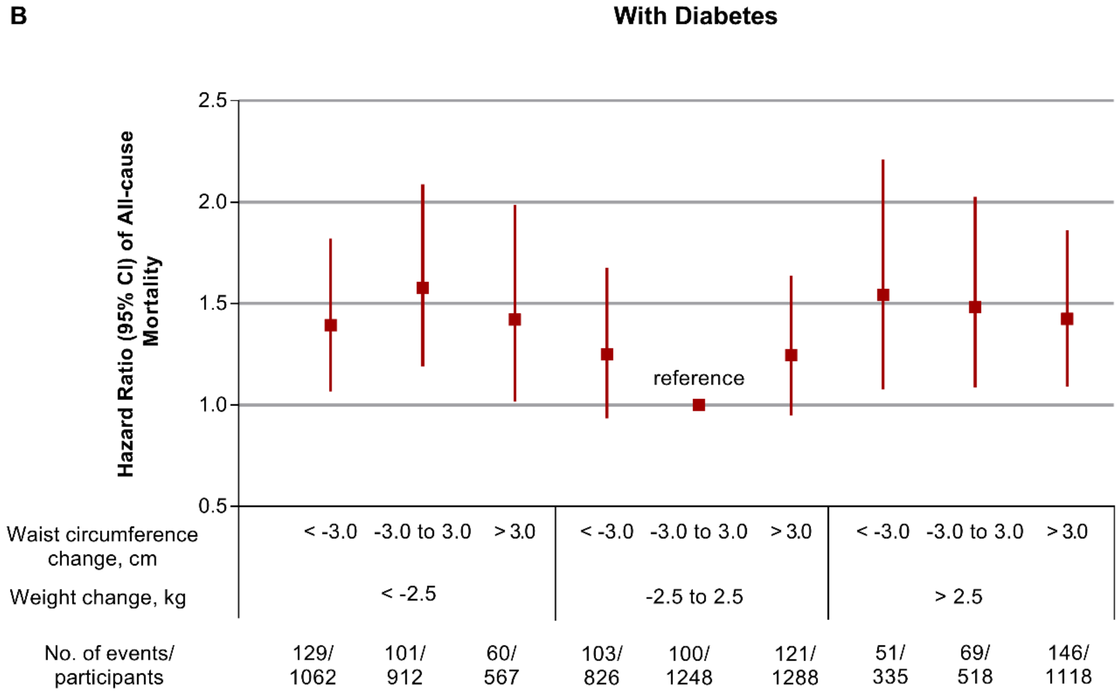

© 2022 Yuan Y et al. *JAMA Network Open.*

eTable 1. Other Variables in the Associations Between Weight Change Categories and All-Cause Mortality.

Other Variables[a]	HRs (95% CI)	*P*
**DFTJ Cohort**		
Weight at cohort recruitment, kg	0.99 (0.97-1.00)	0.0072
Height at cohort recruitment, cm	0.99 (0.97-1.00)	0.14
Waist circumference change, cm	1.00 (0.99-1.01)	0.84
Physical activity, h/week	0.99 (0.98-1.00)	0.075
Smoking status		
Never smoker	1 (reference)	-
Previous smoker	1.08 (0.80-1.45)	0.63
Current smoker	1.52 (1.18-1.96)	0.0013
Alcohol intake		
Never drinker	1 (reference)	-
Previous drinker	1.04 (0.70-1.56)	0.84
Current drinker	1.10 (0.89-1.37)	0.38
Dietary pattern		
Daily FV, weekly but not daily meat	1 (reference)	-
Intermediate pattern	0.84 (0.69-1.03)	0.099
Less than daily FV, daily meat	0.78 (0.58-1.05)	0.11
Educational attainment		
Primary school or below	1 (reference)	-
Middle school	0.84 (0.68-1.03)	0.094
High school or beyond	0.71 (0.57-0.89)	0.0035
Hypertension	1.52 (1.23-1.88)	0.0001
Diabetes	1.32 (1.08-1.61)	0.0065

© 2022 Yuan Y et al. *JAMA Network Open.*

eTable 1. Other Variables in the Associations Between Weight Change Categories and All-Cause Mortality (Continued).

Other Variables[a]	HRs (95% CI)	*P*
**Kailuan Study**		
Weight at cohort recruitment, kg	0.99 (0.99-1.00)	0.0015
Height at cohort recruitment, cm	1.00 (0.99-1.01)	0.96
Waist circumference change, cm	1.00 (1.00-1.00)	0.88
Physical activity		
No physical activity	1 (reference)	-
Occasional physical activity	1.08 (1.00-1.17)	0.055
Regular physical activity	0.84 (0.76-0.94)	0.0016
Smoking status		
Never smoker	1 (reference)	-
Former smoker	1.08 (0.92-1.26)	0.36
Current smoker	1.23 (1.12-1.34)	<0.0001
Alcohol intake		
Never drinker	1 (reference)	-
Former drinker	0.91 (0.63-1.33)	0.64
Current drinker	0.79 (0.72-0.87)	<0.0001
Dietary pattern (based on salt intake, g/day)		
Favorable pattern (<6)	1 (reference)	-
Intermediate pattern (6-9)	0.97 (0.88-1.06)	0.45
Unfavorable pattern (≥10)	1.03 (0.90-1.18)	0.68
Educational attainment		
Primary school or below	1 (reference)	-
Middle school	0.94 (0.86-1.04)	0.23
High school or beyond	0.67 (0.59-0.77)	<0.0001
Hypertension	1.27 (1.18-1.37)	<0.0001
Diabetes	1.76 (1.62-1.91)	<0.0001

[a] The age at risk (5 years) and gender were in the strata of models, therefore there were no risk estimates for these variables.

© 2022 Yuan Y et al. *JAMA Network Open.*

eTable 2. Other Variables in the Associations Between Waist Circumference Change Categories and All-Cause Mortality.

Other Variables[a]	HRs (95% CI)	*P*
**DFTJ Cohort**		
Weight change, kg	0.97 (0.95-0.99)	0.0137
Height at cohort recruitment, cm	0.98 (0.96-0.99)	0.0068
Waist circumference at cohort recruitment, cm	0.99 (0.98-1.00)	0.24
Physical activity, h/week	0.99 (0.98-1.00)	0.071
Smoking status		
Never smoker	1 (reference)	-
Previous smoker	1.08 (0.80-1.45)	0.63
Current smoker	1.56 (1.21-2.01)	0.0007
Alcohol intake		
Never drinker	1 (reference)	-
Previous drinker	1.04 (0.70-1.56)	0.84
Current drinker	1.09 (0.88-1.36)	0.44
Dietary pattern		
Daily FV, weekly but not daily meat	1 (reference)	-
Intermediate pattern	0.84 (0.69-1.03)	0.1
Less than daily FV, daily meat	0.79 (0.59-1.07)	0.13
Educational attainment		
Primary school or below	1 (reference)	-
Middle school	0.84 (0.68-1.03)	0.097
High school or beyond	0.72 (0.57-0.90)	0.0042
Hypertension	1.48 (1.20-1.83)	0.0003
Diabetes	1.32 (1.08-1.61)	0.0068

© 2022 Yuan Y et al. *JAMA Network Open.*

eTable 2. Other Variables in the Associations Between Waist Circumference Change Categories and All-Cause Mortality (Continued).

Other Variables[a]	HRs (95% CI)	*P*
**Kailuan Study**		
Weight change, kg	1.00 (0.99-1.00)	0.29
Height at cohort recruitment, cm	1.00 (0.99-1.00)	0.13
Waist circumference at cohort recruitment, cm	1.00 (1.00-1.01)	0.21
Physical activity		
No physical activity	1 (reference)	-
Occasional physical activity	1.08 (1.00-1.17)	0.056
Regular physical activity	0.85 (0.76-0.94)	0.0024
Smoking status		
Never smoker	1 (reference)	-
Former smoker	1.08 (0.92-1.27)	0.33
Current smoker	1.24 (1.13-1.36)	<0.0001
Alcohol intake		
Never drinker	1 (reference)	-
Former drinker	0.91 (0.62-1.33)	0.63
Current drinker	0.79 (0.72-0.87)	<0.0001
Dietary pattern (based on salt intake, g/day)		
Favorable pattern (<6)	1 (reference)	-
Intermediate pattern (6-9)	0.96 (0.88-1.06)	0.43
Unfavorable pattern (≥10)	1.02 (0.89-1.17)	0.79
Educational attainment		
Primary school or below	1 (reference)	-
Middle school	0.95 (0.86-1.04)	0.25
High school or beyond	0.67 (0.59-0.77)	<0.0001
Hypertension	1.24 (1.15-1.33)	<0.0001
Diabetes	1.73 (1.59-1.88)	<0.0001

[a] The age at risk (5 years) and gender were in the strata of models, therefore there were no risk estimates for these variables.

© 2022 Yuan Y et al. *JAMA Network Open.*

eTable 3. Basic Characteristics of the Participants by the Joint Categories of Weight and Waist Circumference Change.

	Weight Loss (<-2.5 kg)			Weight Stable (-2.5 to 2.5 kg)			Weight Gain (>2.5 kg)		
**Characteristics[a]**	**Waist Loss**	**Waist Stable**	**Waist Gain**	**Waist Loss**	**Waist Stable**	**Waist Gain**	**Waist Loss**	**Waist Stable**	**Waist Gain**
	**(< -3.0 cm)**	**(-3.0 to 3.0 cm)**	**(> 3.0 cm)**	**(< -3.0 cm)**	**(-3.0 to 3.0 cm)**	**(> 3.0 cm)**	**(< -3.0 cm)**	**(-3.0 to 3.0 cm)**	**(> 3.0 cm)**
**DFTJ Cohort, No. of events/participants**	109/1486	73/1477	56/684	26/912	93/2368	80/2197	6/121	18/449	62/1257
Change duration, years	4.6 (0.1)	4.6 (0.1)	4.6 (0.2)	4.6 (0.1)	4.6 (0.1)	4.6 (0.2)	4.6 (0.1)	4.6 (0.1)	4.6 (0.1)
Age at cohort recruitment, years	62.4 (7.3)	62.5 (7.4)	63.1 (7.5)	60.8 (6.7)	61.0 (6.9)	61.5 (7.1)	60.1 (6.5)	59.7 (7.0)	60.8 (7.4)
Men	609 (41.0%)	667 (45.2%)	304 (44.4%)	290 (31.8%)	850 (35.9%)	826 (37.6%)	33 (27.3%)	141 (31.4%)	483 (38.4%)
Women	877 (59.0%)	810 (54.8%)	380 (55.6%)	622 (68.2%)	1518 (64.1%)	1371 (62.4%)	88 (72.7%)	308 (68.6%)	774 (61.6%)
Weight at cohort recruitment, kg	64.8 (10.0)	65.5 (10.3)	65.9 (10.0)	61.0 (9.8)	61.7 (9.6)	62.3 (9.6)	58.5 (10.7)	59.5 (9.6)	60.6 (10.0)
BMI at cohort recruitment [b], kg/m^2	24.9 (3.2)	25.1 (3.2)	25.2 (3.1)	23.9 (3.1)	24.0 (3.0)	24.3 (3.1)	23.0 (3.2)	23.4 (3.1)	23.6 (3.2)
Height at cohort recruitment, cm	161.1 (7.7)	161.4 (7.8)	161.4 (7.6)	159.7 (7.3)	160.0 (7.4)	160.1 (7.5)	159.2 (7.5)	159.4 (7.4)	160.1 (7.3)
Waist circumference at cohort recruitment, cm	87.3 (9.4)	82.7 (8.7)	79.2 (8.5)	87.1 (8.9)	82.2 (8.4)	78.9 (8.1)	87.5 (9.0)	82.0 (8.6)	78.9 (8.5)
Physical activity [c], h/week	9.9 (7.8)	9.5 (7.2)	9.1 (7.1)	9.8 (7.4)	9.5 (7.5)	9.6 (7.3)	9.5 (7.6)	9.3 (7.8)	9.3 (7.5)
Smoking status [c]									
Never smoker	1131 (76.1%)	1099 (74.4%)	498 (72.8%)	728 (79.8%)	1867 (78.8%)	1712 (77.9%)	104 (86.0%)	355 (79.1%)	951 (75.7%)
Previous smoker	122 (8.2%)	148 (10.0%)	75 (11.0%)	70 (7.7%)	183 (7.7%)	201 (9.2%)	7 (5.8%)	26 (5.8%)	138 (11.0%)
Current smoker	228 (15.3%)	225 (15.2%)	106 (15.5%)	111 (12.2%)	311 (13.1%)	274 (12.5%)	9 (7.4%)	65 (14.5%)	166 (13.2%)
Alcohol intake [c]									
Never drinker	1082 (72.8%)	1047 (70.9%)	494 (72.2%)	666 (73.0%)	1732 (73.1%)	1558 (70.9%)	89 (73.6%)	332 (73.9%)	883 (70.3%)
Previous drinker	64 (4.3%)	86 (5.8%)	24 (3.5%)	32 (3.5%)	79 (3.3%)	91 (4.1%)	3 (2.5%)	15 (3.3%)	55 (4.4%)
Current drinker	334 (22.5%)	342 (23.2%)	160 (23.4%)	210 (23.0%)	548 (23.1%)	540 (24.6%)	29 (24.0%)	100 (22.3%)	319 (25.4%)
Dietary pattern [c]									
Daily FV, weekly but not daily meat	396 (26.7%)	372 (25.2%)	179 (26.2%)	244 (26.8%)	592 (25.0%)	528 (24.0%)	32 (26.5%)	99 (22.1%)	309 (24.6%)
Intermediate pattern	893 (60.1%)	889 (60.2%)	417 (61.0%)	531 (58.2%)	1446 (61.1%)	1403 (63.9%)	71 (58.7%)	267 (59.5%)	760 (60.5%)
Less than daily FV, daily meat	184 (12.4%)	203 (13.7%)	81 (11.8%)	130 (14.3%)	308 (13.0%)	245 (11.2%)	17 (14.1%)	74 (16.5%)	171 (13.6%)
Educational attainment [c]									
Primary school or below	444 (29.9%)	387 (26.2%)	199 (29.1%)	264 (29.0%)	585 (24.7%)	606 (27.6%)	35 (28.9%)	104 (23.2%)	284 (22.6%)
Middle school	564 (38.0%)	562 (38.1%)	253 (37.0%)	344 (37.7%)	916 (38.7%)	836 (38.1%)	48 (39.7%)	185 (41.2%)	502 (39.9%)
High school or beyond	467 (31.4%)	520 (35.2%)	223 (32.6%)	297 (32.6%)	852 (36.0%)	747 (34.0%)	34 (28.1%)	159 (35.4%)	462 (36.8%)
Hypertension	943 (63.5%)	927 (62.8%)	439 (64.2%)	548 (60.1%)	1449 (61.2%)	1390 (63.3%)	76 (62.8%)	257 (57.2%)	826 (65.7%)
Diabetes	336 (22.6%)	372 (25.2%)	148 (21.6%)	125 (13.7%)	396 (16.7%)	363 (16.5%)	20 (16.5%)	76 (16.9%)	229 (18.2%)
**Kailuan Study, No. of events/participants**	420/4328	277/3476	252/3014	432/5575	415/7460	547/8100	250/2885	299/4184	613/8159
Change duration, years	4.1 (0.5)	4.0 (0.5)	4.0 (0.6)	4.1 (0.5)	4.1 (0.5)	4.1 (0.5)	4.1 (0.4)	4.1 (0.4)	4.1 (0.5)
Age at cohort recruitment, years	53.9 (9.5)	52.3 (8.9)	52.1 (8.8)	53.5 (9.1)	52.2 (8.8)	52.4 (8.6)	53.6 (9.4)	52.8 (9.0)	53.2 (8.8)
Men	3359 (77.6%)	2834 (81.5%)	2487 (82.5%)	4235 (76.0%)	5739 (76.9%)	6386 (78.8%)	2154 (74.7%)	3173 (75.8%)	6296 (77.2%)
Women	969 (22.4%)	642 (18.5%)	527 (17.5%)	1340 (24.0%)	1721 (23.1%)	1714 (21.2%)	731 (25.3%)	1011 (24.2%)	1863 (22.8%)
Weight at cohort recruitment, kg	75.0 (10.6)	74.7 (10.2)	76.1 (10.8)	69.5 (10.5)	69.9 (10.2)	70.3 (10.4)	66.3 (10.6)	67.5 (10.4)	67.8 (10.5)

© 2022 Yuan Y et al. *JAMA Network Open.*

BMI at cohort recruitment[b], kg/m^2	26.7 (3.5)	26.4 (3.3)	26.8 (3.4)		25.0 (3.2)	25.1 (3.0)	25.1 (3.1)		23.9 (3.4)	24.2 (3.1)	24.2 (3.2)
Height at cohort recruitment, cm	167.5 (7.0)	168.1 (6.7)	168.6 (6.7)		166.5 (7.0)	166.8 (7.0)	167.3 (7.0)		166.4 (7.1)	166.8 (7.0)	167.2 (6.9)
Waist circumference at cohort recruitment, cm	93.7 (8.7)	88.2 (8.0)	84.5 (8.0)		92.5 (9.0)	87.3 (8.3)	83.3 (8.2)		93.7 (9.4)	87.4 (8.6)	83.2 (8.5)
Physical activity [c]											
No physical activity	1211 (28.0%)	1148 (33.0%)	823 (27.3%)		1672 (30.0%)	2490 (33.4%)	2407 (29.7%)		837 (29.0%)	1298 (31.0%)	2320 (28.4%)
Occasional physical activity	2466 (57.0%)	1815 (52.2%)	1722 (57.1%)		3111 (55.8%)	3756 (50.4%)	4305 (53.2%)		1690 (58.6%)	2262 (54.1%)	4404 (54.0%)
Regular physical activity	642 (14.8%)	505 (14.5%)	469 (15.6%)		786 (14.1%)	1203 (16.1%)	1378 (17.0%)		356 (12.3%)	618 (14.8%)	1423 (17.4%)
Smoking status [c]											
Never smoker	2887 (66.7%)	2101 (60.4%)	1903 (63.1%)		3659 (65.6%)	4550 (61.0%)	4975 (61.4%)		1935 (67.1%)	2695 (64.4%)	5190 (63.6%)
Former smoker	179 (4.1%)	151 (4.3%)	130 (4.3%)		227 (4.1%)	327 (4.4%)	367 (4.5%)		101 (3.5%)	204 (4.9%)	430 (5.3%)
Current smoker	1254 (29.0%)	1216 (35.0%)	980 (32.5%)		1684 (30.2%)	2573 (34.5%)	2747 (33.9%)		847 (29.4%)	1279 (30.6%)	2527 (31.0%)
Alcohol intake [c]											
Never drinker	3141 (72.6%)	2295 (66.0%)	2047 (67.9%)		3912 (70.2%)	4745 (63.6%)	5345 (66.0%)		2074 (71.9%)	2800 (66.9%)	5518 (67.6%)
Former drinker	36 (0.8%)	12 (0.4%)	16 (0.5%)		28 (0.5%)	46 (0.6%)	51 (0.6%)		6 (0.2%)	33 (0.8%)	46 (0.6%)
Current drinker	1143 (26.4%)	1159 (33.3%)	951 (31.6%)		1628 (29.2%)	2657 (35.6%)	2693 (33.3%)		802 (27.8%)	1344 (32.1%)	2580 (31.6%)
Dietary pattern (based on salt intake, g/day) [c]											
Favorable pattern (<6)	660 (15.3%)	626 (18.0%)	563 (18.7%)		902 (16.2%)	1285 (17.2%)	1474 (18.2%)		468 (16.2%)	617 (14.8%)	1274 (15.6%)
Intermediate pattern (6-9)	3347 (77.3%)	2495 (71.8%)	2154 (71.5%)		4220 (75.7%)	5375 (72.1%)	5690 (70.3%)		2174 (75.4%)	3161 (75.6%)	5907 (72.4%)
Unfavorable pattern (≥10)	313 (7.2%)	346 (10.0%)	296 (9.8%)		447 (8.0%)	787 (10.6%)	924 (11.4%)		241 (8.4%)	399 (9.5%)	965 (11.8%)
Educational attainment [c]											
Primary school or below	381 (8.8%)	280 (8.1%)	262 (8.7%)		493 (8.8%)	581 (7.8%)	729 (9.0%)		276 (9.6%)	359 (8.6%)	840 (10.3%)
Middle school	3183 (73.5%)	2539 (73.0%)	2240 (74.3%)		4085 (73.3%)	5302 (71.1%)	5912 (73.0%)		2177 (75.5%)	2971 (71.0%)	5947 (72.9%)
High school or beyond	756 (17.5%)	644 (18.5%)	503 (16.7%)		991 (17.8%)	1564 (21.0%)	1437 (17.7%)		428 (14.8%)	846 (20.2%)	1351 (16.6%)
Hypertension	2193 (50.7%)	1721 (49.5%)	1500 (49.8%)		2767 (49.6%)	3718 (49.8%)	4043 (49.9%)		1457 (50.5%)	2145 (51.3%)	4399 (53.9%)
Diabetes	726 (16.8%)	540 (15.5%)	419 (13.9%)		701 (12.6%)	852 (11.4%)	925 (11.4%)		315 (10.9%)	442 (10.6%)	889 (10.9%)

Abbreviations: BMI, body mass index; DFTJ, Dongfeng-Tongji; FV, fruit and vegetables.

[a] Values are presented as mean (SD) or n (%).

[b] Body-mass index (BMI) is weight in kilograms divided by the height in meters squared.

[c] Data were incomplete for these variables. In the DFTJ cohort, 0.2% (n=23), 0.4% (n=41), 0.3% (n=37), 1.0% (n=110), and 0.7% (n=72) of participants had missing data for physical activity, smoking status, alcohol intake, dietary pattern, and educational attainment, respectively. In the Kailuan study, 0.1% (n=64), 0.1% (n=63), 0.2% (n=73), 0.2% (n=71), and 0.2% (n=104) of participants had missing data for physical activity, smoking status, alcohol intake, dietary pattern, and educational attainment, respectively. The other variables included in the analyses did not have missing data.

© 2022 Yuan Y et al. *JAMA Network Open.*

eTable 4. Associations Between Weight Change Categories and All-Cause Mortality Among Never Smokers.

**Variable**[a]	**Weight change, kg**				
	**Loss**		**Stable**		**Gain**
	**(< -2.5)**		**(-2.5≤change≤2.5)**		**(> 2.5)**
**DFTJ Cohort**					
No. of events/person years	139/15 222		136/24 176		57/7888
HR (95% CI)	1.37 (1.06-1.76)		1 (reference)		1.32 (0.96-1.82)
**Kailuan Study**					
No. of events/person years	656/52 746		922/102 039		757/75 201
HR (95% CI)	1.31 (1.18-1.45)		1 (reference)		1.03 (0.93-1.14)
**Pooled Results**					
No. of events/person years	795/67 968		1058/126 215		814/83 089
HR (95% CI)	1.32 (1.20-1.45)		1 (reference)		1.05 (0.96-1.16)

[a]The multivariable models were adjusted for height and weight at cohort recruitment, waist circumference change (continuous variables), alcohol intake status, dietary pattern, educational attainment, physical activity, hypertension, and diabetes, and stratified by age at risk (5-year interval) and sex. We conducted cohort-specific analyses, which were pooled together using fixed-effect meta-analyses.

© 2022 Yuan Y et al. *JAMA Network Open.*

eTable 5. Associations Between Waist Circumference Change Categories and All-Cause Mortality Among Never Smokers.

Variable[a]	Waist circumference change, cm				
	Loss		Stable		Gain
	(< 3.0)		(-3.0≤change≤3.0)		(> 3.0)
**DFTJ Cohort**					
No. of events/person years	88/10 969		121/18 640		123/17 677
HR (95% CI)	1.14 (0.85-1.53)		1 (reference)		1.02 (0.78-1.33)
**Kailuan Study**					
No. of events/person years	771/64 710		644/72 305		920/92 971
HR (95% CI)	1.11 (0.99-1.24)		1 (reference)		1.12 (1.01-1.25)
**Pooled Results**					
No. of events/person years	859/75 679		765/90 945		1043/110 648
HR (95% CI)	1.11 (1.00-1.23)		1 (reference)		1.11 (1.01-1.22)

[a]The multivariable models were adjusted for height and waist circumference at cohort recruitment, weight change (continuous variable), alcohol intake status, dietary pattern, educational attainment, physical activity, hypertension, and diabetes, and stratified by age at risk (5-year interval) and sex. We conducted cohort-specific analyses, which were pooled together using fixed-effect meta-analyses.

© 2022 Yuan Y et al. *JAMA Network Open.*

eTable 6. Associations Between Weight Change Categories and All-Cause Mortality (Random-effects Meta-analyses).

Variable[a]	Weight change, kg		
	Loss (< -2.5)	Stable (-2.5 ≤ change ≤ 2.5)	Gain (> 2.5)
**DFTJ Cohort**			
No. of events/person years	238/20 234	199/30 695	86/10 218
HR (95% CI)	1.56 (1.28-1.91)	1 (reference)	1.30 (1.00-1.69)
**Kailuan Study**			
No. of events/person years	949/83 503	1394/164 441	1162/116 981
HR (95% CI)	1.29 (1.18-1.40)	1 (reference)	1.09 (1.00-1.18)
**Pooled Results**			
No. of events/person years	1187/103 737	1593/195 136	1248/127 199
HR (95% CI)	1.39 (1.15-1.67)	1 (reference)	1.14 (0.97-1.33)

[a] The multivariable models were adjusted for height and weight at cohort recruitment, waist circumference change (continuous variables), smoking status, alcohol intake status, dietary pattern, educational attainment, physical activity, hypertension, and diabetes, and stratified by age at risk (5-year interval) and sex. We conducted cohort-specific analyses, which were pooled together using random-effects meta-analyses.

© 2022 Yuan Y et al. *JAMA Network Open.*

eTable 7. Associations Between Waist Circumference Change Categories and All-Cause Mortality (Random-effects Meta-analyses).

Variable[a]	Waist circumference change, cm				
	Loss (< -3.0)		Stable (-3.0 ≤ change ≤ 3.0)		Gain (> 3.0)
**DFTJ Cohort**					
No. of events/person years	141/14 017		184/24 032		198/23 098
HR (95% CI)	1.22 (0.97-1.54)		1 (reference)		1.14 (0.92-1.41)
**Kailuan Study**					
No. of events/person years	1102/98 141		991/117 698		1412/149 086
HR (95% CI)	1.13 (1.03-1.23)		1 (reference)		1.11 (1.02-1.21)
**Pooled Results**					
No. of events/person years	1243/112 158		1175/141 730		1610/172 184
HR (95% CI)	1.14 (1.05-1.24)		1 (reference)		1.11 (1.03-1.21)

[a] The multivariable models were adjusted for height and waist circumference at cohort recruitment, weight change (continuous variables) smoking status, alcohol intake status, dietary pattern, educational attainment, physical activity, hypertension, and diabetes, and stratified by age at risk (5-year interval) and sex. We conducted cohort-specific analyses, which were pooled together using random-effects meta-analyses.

© 2022 Yuan Y et al. *JAMA Network Open.*

eTable 8. Associations of Baseline Characteristics With Weight Change and Waist Circumference Change.

Characteristics [a]	Weight change, kg			Waist circumference change, cm	
	β (95% CI)	P value		β (95% CI)	P value
**DFTJ Cohort**					
Age at cohort recruitment, 5 years	-0.31 (-0.37, -0.25) [b]	<0.0001		0.48 (0.40, 0.57)	<0.0001
Sex					
Men	Ref			Ref	
Women	-0.47 (-0.73, -0.20)	0.0005		0.02 (-0.35, 0.40)	0.91
Weight at cohort recruitment, kg	-0.12 (-0.13, -0.10)	<0.0001		0.57 (0.55, 0.59)	<0.0001
Height at cohort recruitment, cm	0.03 (0.01, 0.04)	0.0015		-0.25 (-0.27, -0.22)	<0.0001
Waist circumference at cohort recruitment, cm	0.02 (0.01, 0.04)	0.0004		-0.75 (-0.77, -0.73)	<0.0001
Physical activity [c], h/week	0.02 (0.00, 0.03)	0.0146		0.00 (-0.02, 0.02)	0.97
Smoking status [c]					
Never smoker	Ref			Ref	
Previous smoker	-0.04 (-0.36, 0.29)	0.82		-0.08 (-0.54, 0.38)	0.73
Current smoker	-0.06 (-0.32, 0.21)	0.67		0.49 (0.11, 0.87)	0.0117
Alcohol intake [c]					
Never drinker	Ref			Ref	
Previous drinker	0.01 (-0.42, 0.43)	0.98		0.13 (-0.48, 0.73)	0.68
Current drinker	0.15 (-0.07, 0.37)	0.18		0.38 (0.06, 0.69)	0.0182
Dietary pattern [c]					
Daily FV, weekly but not daily meat	Ref			Ref	
Intermediate pattern	-0.04 (-0.23, 0.16)	0.71		0.08 (-0.19, 0.35)	0.57
Less than daily FV, daily meat	-0.37 (-1.60, 0.86)	0.55		0.16 (-1.59, 1.90)	0.86
Educational attainment [c]					
Primary school or below	Ref			Ref	
Middle school	-0.07 (-0.26, 0.12)	0.46		-0.42 (-0.69, -0.14)	0.0029
High school or beyond	-0.04 (-0.24, 0.17)	0.73		-0.80 (-1.08, -0.51)	<0.0001
Hypertension					
No	Ref			Ref	
Yes	-0.03 (-0.19, 0.13)	0.71		0.08 (-0.15, 0.30)	0.49

© 2022 Yuan Y et al. *JAMA Network Open.*

Diabetes					
No	Ref			Ref	
Yes	-0.63 (-0.85, -0.42)	<0.0001		0.21 (-0.10, 0.51)	0.18
**Kailuan Study**					
Age at cohort recruitment, 5 years	-0.09 (-0.12, -0.06) [b]	<0.0001		0.61 (0.57, 0.66)	<0.0001
Sex					
Men	Ref			Ref	
Women	-0.20 (-0.36, -0.04)	0.0123		-0.19 (-0.41, 0.04)	0.10
Weight at cohort recruitment, kg	-0.26 (-0.26, -0.25)	<0.0001		0.45 (0.44, 0.46)	<0.0001
Height at cohort recruitment, cm	0.12 (0.11, 0.13)	<0.0001		-0.07 (-0.08, -0.06)	<0.0001
Waist circumference at cohort recruitment, cm	0.09 (0.08, 0.10)	<0.0001		-0.82 (-0.83, -0.81)	<0.0001
Physical activity [c]					
Regular physical activity	Ref			Ref	
Occasional physical activity	-0.47 (-0.62, -0.32)	<0.0001		0.01 (-0.20, 0.23)	0.89
No physical activity	-0.16 (-0.39, 0.06)	0.15		0.29 (-0.03, 0.60)	0.07
Smoking status [c]					
Never smoker	Ref			Ref	
Previous smoker	0.03 (-0.21, 0.27)	0.81		-0.16 (-0.50, 0.19)	0.37
Current smoker	0.07 (-0.08, 0.21)	0.36		0.22 (0.02, 0.41)	0.0347
Alcohol intake [c]					
Never drinker	Ref			Ref	
Previous drinker	0.08 (-0.22, 0.39)	0.59		-0.12 (-0.54, 0.31)	0.59
Current drinker	0.15 (0.01, 0.29)	0.0319		0.19 (-0.01, 0.38)	0.06
Dietary pattern (based on salt intake, g/day) [c]					
Favorable pattern (<6)	Ref			Ref	
Intermediate pattern (6-9)	0.17 (-0.01, 0.35)	0.07		0.28 (0.02, 0.53)	0.0343
Unfavorable pattern (≥10)	0.59 (0.36, 0.82)	<0.0001		0.51 (0.18, 0.83)	0.0024
Educational attainment [c]					
Primary school or below	Ref			Ref	
Middle school	-0.33 (-0.51, -0.15)	0.0004		0.21 (-0.05, 0.47)	0.11
High school or beyond	-0.32 (-0.53, -0.11)	0.0033		-0.56 (-0.86, -0.27)	0.0002
Hypertension					

© 2022 Yuan Y et al. *JAMA Network Open.*

No	Ref			Ref	
Yes	-0.02 (-0.12, 0.08)	0.68		0.31 (0.16, 0.45)	<0.0001
Diabetes					
No	Ref			Ref	
Yes	-0.43 (-0.60, -0.27)	<0.0001		0.23 (-0.01, 0.47)	0.06

[a] The generalized linear models included age (each 5 years increase), sex, weight, height, and waist circumference, smoking status, alcohol intake status, dietary pattern, educational attainment, physical activity, hypertension, and diabetes status. All these independent variables in the models were collected at baseline.

[b] The risk estimates were highlighted with red color and bold font if the corresponding P value was less than 0.05 (statistical significance).

[c] Data were incomplete for these variables. For the categorical variable, the incomplete data was imputed as a missing category in the analyses. For the continuous variable, the incomplete data was imputed as the median value, in addition with an indicator variable to denote the missing data. In the Kailuan study, 4.2% (n=1961), 3.1% (n=1471), 3.1% (n=1447), 4.1% (n=1919), and 3.9% (n=1825) of participants had missing data for physical activity, smoking status, alcohol intake, dietary pattern, and educational attainment, respectively. The other variables included in the analyses did not have missing data.

© 2022 Yuan Y et al. *JAMA Network Open.*

eTable 9. Associations of Baseline Characteristics With Joint Changes in Weight and Waist Circumference.

Characteristics[a]	Weight Loss (<-2.5 kg)				Weight Stable (-2.5 to 2.5 kg)				Weight Gain (>2.5 kg)		
	Waist Loss	Waist Stable	Waist Gain		Waist Loss	Waist Stable	Waist Gain		Waist Loss	Waist Stable	Waist Gain
	(< -3.0 cm)	(-3.0 to 3.0 cm)	(> 3.0 cm)		(< -3.0 cm)	(-3.0 to 3.0 cm)	(> 3.0 cm)		(< -3.0 cm)	(-3.0 to 3.0 cm)	(> 3.0 cm)
**DFTJ Cohort, No. of participants**	1486 (13.6%)	1477 (13.5%)	684 (6.2%)		912 (8.3%)	2368 (21.6%)	2197 (20.1%)		121 (1.1%)	449 (4.1%)	1257 (11.5%)
Age at cohort recruitment, 5 years	1.01 (0.96, 1.07)	1.25 (1.18, 1.32) [b]	1.55 (1.44, 1.67)		0.83 (0.78, 0.98)	-	1.21 (1.15, 1.28)		0.73 (0.62, 0.85)	0.82 (0.76, 0.90)	1.08 (1.02, 1.14)
Sex						-					
Men	Ref	Ref	Ref		Ref	-	Ref		Ref	Ref	Ref
Women	1.02 (0.81, 1.29)	1.03 (0.81, 1.29)	1.32 (0.96, 1.81)		0.98 (0.73, 1.32)	-	0.98 (0.79, 1.22)		0.65 (0.33, 1.31)	0.93 (0.64, 1.37)	0.87 (0.68, 1.12)
Weight at cohort recruitment, kg	0.93 (0.92, 0.95)	1.16 (1.14, 1.18)	1.38 (1.35, 1.41)		0.82 (0.81, 0.84)	-	1.21 (1.19, 1.23)		0.76 (0.73, 0.79)	0.91 (0.89, 0.93)	1.10 (1.08, 1.12)
Height at cohort recruitment, cm	1.04 (1.03, 1.06)	0.95 (0.94, 0.97)	0.89 (0.87, 0.91)		1.08 (1.06, 1.10)	-	0.93 (0.92, 0.94)		1.12 (1.08, 1.16)	1.03 (1.01, 1.06)	0.96 (0.95, 0.98)
Waist circumference at cohort recruitment, cm	1.13 (1.11, 1.15)	0.88 (0.86, 0.89)	0.71 (0.69, 0.73)		1.25 (1.23, 1.27)	-	0.80 (0.79, 0.82)		1.32 (1.28, 1.37)	1.09 (1.07, 1.11)	0.88 (0.86, 0.89)
Physical activity [c], h/week	0.99 (0.98, 1.00)	0.99 (0.98, 1.01)	0.99 (0.98, 1.01)		1.00 (0.99, 1.02)	-	1.00 (0.99, 1.01)		1.00 (0.96, 1.03)	1.00 (0.99, 1.02)	1.00 (0.99, 1.01)
Smoking status [c]						-					
Never smoker	Ref	Ref	Ref		Ref	-	Ref		Ref	Ref	Ref
Previous smoker	0.83 (0.62, 1.10)	0.89 (0.68, 1.17)	1.13 (0.79, 1.63)		0.96 (0.67, 1.40)	-	0.77 (0.59, 1.00)		0.89 (0.34, 2.34)	0.89 (0.54, 1.47)	0.72 (0.53, 0.99)
Current smoker	0.99 (0.79, 1.26)	1.14 (0.90, 1.44)	1.38 (1.01, 1.90)		0.95 (0.70, 1.28)	-	1.06 (0.85, 1.32)		0.62 (0.29, 1.34)	1.26 (0.86, 1.85)	1.24 (0.96, 1.58)
Alcohol intake [c]						-					
Never drinker	Ref	Ref	Ref		Ref	-	Ref		Ref	Ref	Ref
Previous drinker	1.20 (0.83, 1.74)	1.33 (0.92, 1.91)	0.83 (0.48, 1.44)		1.13 (0.69, 1.85)	-	1.16 (0.81, 1.67)		0.33 (0.04, 2.53)	2.03 (1.20, 3.42)	1.25 (0.83, 1.88)
Current drinker	0.83 (0.68, 1.00)	0.90 (0.74, 1.10)	1.08 (0.83, 1.40)		1.00 (0.79, 1.28)	-	1.00 (0.84, 1.20)		0.89 (0.49, 1.61)	0.89 (0.65, 1.21)	1.11 (0.91, 1.36)
Dietary pattern [c]						-					
Daily FV, weekly but not daily meat	Ref	Ref	Ref		Ref	-	Ref		Ref	Ref	Ref
Intermediate pattern	1.13 (0.95, 1.34)	0.89 (0.75, 1.06)	1.14 (0.91, 1.44)		0.95 (0.77, 1.18)	-	1.06 (0.90, 1.24)		0.62 (0.37, 1.05)	1.28 (0.98, 1.68)	1.06 (0.89, 1.27)
Less than daily FV, daily meat	1.33 (0.49, 3.61)	0.85 (0.29, 2.47)	0.84 (0.20, 3.53)		0.56 (0.11, 2.91)	-	0.94 (0.35, 2.53)		-	-	0.77 (0.23, 2.57)
Educational attainment [c]						-					
Primary school or below	Ref	Ref	Ref		Ref	-	Ref		Ref	Ref	Ref
Middle school	1.04 (0.87, 1.23)	1.05 (0.88, 1.24)	0.86 (0.68, 1.09)		1.20 (0.97, 1.48)	-	0.85 (0.73, 1.00)		0.93 (0.71, 1.21)	0.93 (0.71, 1.21)	1.01 (0.84, 1.21)
High school or beyond	0.99 (0.83, 1.18)	0.96 (0.80, 1.14)	0.67 (0.52, 0.85)		1.15 (0.92, 1.43)	-	0.73 (0.62, 0.85)		1.00 (0.76, 1.31)	1.00 (0.76, 1.31)	0.90 (0.75, 1.09)
Hypertension						-					
No	Ref	Ref	Ref		Ref	-	Ref		Ref	Ref	Ref
Yes	1.03 (0.89, 1.18)	0.99 (0.86, 1.14)	0.98 (0.81, 1.19)		0.96 (0.81, 1.14)		0.81 (0.54, 1.21)		0.83 (0.67, 1.03)	0.83 (0.67, 1.03)	0.98 (0.85, 1.13)
Diabetes											
No	Ref	Ref	Ref		Ref	-	Ref		Ref	Ref	Ref
Yes	1.32 (1.10, 1.59)	1.79 (1.49, 2.14)	1.58 (1.22, 2.04)		0.70 (0.55, 0.91)		0.99 (0.58, 1.71)		0.96 (0.70, 1.32)	0.96 (0.70, 1.32)	1.17 (0.95, 1.45)
**Kailuan Study, No. of participants**	4328 (9.2%)	3476 (7.4%)	3014 (6.4%)		5575 (11.8%)	7460 (15.8%)	8100 (17.2%)		2885 (6.1%)	4184 (8.9%)	8159 (17.3%)
Age at cohort recruitment, 5 years	1.03 (1.00, 1.05)[b]	1.09 (1.06, 1.12)	1.22 (1.18, 1.26)		0.94 (0.92, 0.97)	-	1.16 (1.14, 1.19)		0.91 (0.89, 0.94)	0.99 (0.97, 1.02)	1.17 (1.14, 1.19)
Sex						-					
Men	Ref	Ref	Ref		Ref	-	Ref		Ref	Ref	Ref
Women	1.16 (1.03, 1.32)	0.88 (0.77, 1.01)	0.86 (0.74, 1.00)		0.99 (0.88, 1.11)	-	0.89 (0.80, 0.99)		0.94 (0.81, 1.09)	1.08 (0.95, 1.22)	1.03 (0.92, 1.14)
Weight at cohort recruitment, kg	0.99 (0.99, 1.00)	1.10 (1.10, 1.11)	1.21 (1.20, 1.22)		0.90 (0.90, 0.91)	-	1.11 (1.11, 1.12)		0.84 (0.84, 0.85)	0.93 (0.92, 0.94)	1.04 (1.04, 1.05)

© 2022 Yuan Y et al. *JAMA Network Open.*

Height at cohort recruitment, cm	1.01 (1.00, 1.01)	0.97 (0.97, 0.98)	0.96 (0.95, 0.97)		1.03 (1.02, 1.04)	-	0.98 (0.97, 0.98)		1.06 (1.05, 1.07)	1.04 (1.04, 1.05)	1.02 (1.01, 1.02)
Waist circumference at cohort recruitment, cm	1.10 (1.09, 1.11)	0.92 (0.92, 0.93)	0.81 (0.80, 0.81)		1.17 (1.16, 1.18)	-	0.85 (0.85, 0.86)		1.23 (1.22, 1.24)	1.06 (1.05, 1.07)	0.90 (0.89, 0.90)
Physical activity [c]											
Regular physical activity	Ref	Ref	Ref		Ref	-	Ref		Ref	Ref	Ref
Occasional physical activity	1.13 (1.00, 1.28)	1.21 (1.06, 1.37)	1.18 (1.02, 1.36)		0.86 (0.77, 0.96)		0.96 (0.87, 1.06)		0.71 (0.62, 0.82)	1.02 (0.90, 1.15)	0.90 (0.82, 1.00)
No physical activity	0.91 (0.75, 1.10)	1.15 (0.96, 1.39)	0.95 (0.76, 1.17)		0.78 (0.66, 0.92)		0.98 (0.85, 1.13)		0.62 (0.49, 0.78)	1.02 (0.86, 1.21)	0.98 (0.85, 1.12)
Smoking status [c]						-					
Never smoker	Ref	Ref	Ref		Ref	-	Ref		Ref	Ref	Ref
Previous smoker	1.03 (0.86, 1.23)	0.92 (0.76, 1.12)	0.88 (0.70, 1.10)		1.02 (0.86, 1.22)	-	0.91 (0.78, 1.07)		1.13 (0.89, 1.43)	0.95 (0.79, 1.15)	0.91 (0.78, 1.07)
Current smoker	0.92 (0.82, 1.03)	1.04 (0.93, 1.17)	1.00 (0.88, 1.13)		1.01 (0.91, 1.13)	-	1.09 (1.00, 1.20)		1.11 (0.96, 1.27)	1.00 (0.89, 1.11)	1.09 (1.00, 1.20)
Alcohol intake [c]						-					
Never drinker	Ref	Ref	Ref		Ref	-	Ref		Ref	Ref	Ref
Previous drinker	0.70 (0.55, 0.89)	0.86 (0.67, 1.09)	0.91 (0.69, 1.19)		0.93 (0.75, 1.16)	-	0.87 (0.71, 1.06)		0.94 (0.70, 1.25)	0.77 (0.60, 0.98)	0.81 (0.67, 0.99)
Current drinker	0.71 (0.64, 0.80)	0.76 (0.68, 0.85)	0.77 (0.68, 0.88)		0.88 (0.80, 0.98)	-	0.89 (0.82, 0.98)		0.80 (0.70, 0.92)	0.91 (0.82, 1.01)	0.82 (0.75, 0.89)
Dietary pattern (based on salt intake, g/day) [c]						-					
Favorable pattern (<6)	Ref	Ref	Ref		Ref	-	Ref		Ref	Ref	Ref
Intermediate pattern (6-9)	1.10 (0.95, 1.27)	1.01 (0.87, 1.16)	1.25 (1.05, 1.48)		1.07 (0.94, 1.22)	-	1.16 (1.04, 1.31)		1.21 (1.01, 1.45)	1.22 (1.06, 1.41)	1.23 (1.10, 1.38)
Unfavorable pattern (≥10)	1.02 (0.84, 1.22)	0.93 (0.77, 1.12)	1.06 (0.85, 1.32)		1.12 (0.94, 1.32)	-	1.16 (1.00, 1.35)		1.20 (0.95, 1.52)	1.34 (1.12, 1.60)	1.38 (1.19, 1.60)
Educational attainment [c]						-					
Primary school or below	Ref	Ref	Ref		Ref	-	Ref		Ref	Ref	Ref
Middle school	1.02 (0.88, 1.17)	1.15 (0.98, 1.34)	1.10 (0.92, 1.31)		0.99 (0.87, 1.14)	-	1.05 (0.93, 1.19)		0.81 (0.69, 0.96)	0.97 (0.84, 1.12)	1.02 (0.90, 1.14)
High school or beyond	0.89 (0.75, 1.05)	0.99 (0.83, 1.18)	0.75 (0.61, 0.92)		0.94 (0.81, 1.10)	-	0.84 (0.73, 0.96)		0.68 (0.56, 0.83)	0.90 (0.77, 1.06)	0.76 (0.66, 0.87)
Hypertension						-					
No	Ref	Ref	Ref		Ref	-	Ref		Ref	Ref	Ref
Yes	0.93 (0.85, 1.00)	0.86 (0.79, 0.94)	0.81 (0.74, 0.89)		1.05 (0.97, 1.13)		0.92 (0.86, 0.99)		1.11 (1.00, 1.22)	1.03 (0.95, 1.12)	0.97 (0.91, 1.04)
Diabetes											
No	Ref	Ref	Ref		Ref	-	Ref		Ref	Ref	Ref
Yes	0.76 (0.68, 0.86)	0.70 (0.62, 0.80)	0.66 (0.57, 0.76)		1.06 (0.93, 1.20)		0.84 (0.75, 0.95)		1.18 (1.00, 1.40)	1.14 (0.99, 1.31)	0.96 (0.85, 1.08)

[a] The multinomial logistic regression model included age (each 5 years increase), sex, weight, height, and waist circumference, smoking status, alcohol intake status, dietary pattern, educational attainment, physical activity, hypertension, and diabetes status. All these independent variables in the model were collected at baseline. The weight and waist change stable group (-2.5 to 2.5 kg, -3 to 3 cm) was set as control group in the multinomial logistic regression analyses.

[b] The adjusted odds ratio was highlighted with red color if the corresponding P value was less than 0.05 (statistical significance).

[c] Data were incomplete for these variables. For the categorical variable, the incomplete data was imputed as a missing category in the analyses. For the continuous variable, the incomplete data was imputed as the median value, in addition with an indicator variable to denote the missing data. In the DFTJ cohort, 12.8% (n=1398), 0.6% (n=71), 0.1% (n=12), 35.7% (n=3910), and 0.8% (n=83) of participants had missing data for physical activity, smoking status, alcohol intake, dietary pattern, and educational attainment, respectively. In the Kailuan study, 4.2% (n=1961), 3.1% (n=1471), 3.1% (n=1447), 4.1% (n=1919), and 3.9% (n=1825) of participants had missing data for physical activity, smoking status, alcohol intake, dietary pattern, and educational attainment, respectively. The other variables included in the analyses did not have missing data.

© 2022 Yuan Y et al. *JAMA Network Open.*

eReferences

1. Eckel RH, Jakicic JM, Ard JD, et al. 2013 AHA/ACC guideline on lifestyle management to reduce cardiovascular risk: a report of the American College of Cardiology/American Heart Association Task Force on Practice Guidelines. *J Am Coll Cardiol.* 2014;63(25 Pt B):2960-2984.
2. Lv J, Yu C, Guo Y, et al. Adherence to healthy lifestyle and cardiovascular diseases in the Chinese population. *J Am Coll Cardiol.* 2017;69(9):1116-1125.
3. Wu S, An S, Li W, et al. Association of trajectory of cardiovascular health score and incident cardiovascular disease. *JAMA Netw Open.* 2019;2(5):e194758.
4. Zheng Y, Manson JE, Yuan C, et al. Associations of Weight Gain From Early to Middle Adulthood With Major Health Outcomes Later in Life. *JAMA*. 2017;318(3):255-269.
5. Hong LS. Lexis expansion-age-at-risk adjustment for survival analysis. 2013. https://www.lexjansen.com/phuse/2013/sp/SP09.pdf. Accessed January 30, 2020.
6. Di Angelantonio E, Bhupathiraju ShN, Wormser D, et al. Global BMI Mortality Collaboration. Body-mass index and all-cause mortality: individual-participant-data meta-analysis of 239 prospective studies in four continents. *Lancet.* 2016;388(10046):776-786.

© 2022 Yuan Y et al. *JAMA Network Open.*
